# A rare population of tumor antigen-specific CD4^+^CD8^+^ double-positive αβ T lymphocytes uniquely provide CD8-independent TCR genes for engineering therapeutic T cells

**DOI:** 10.1186/s40425-018-0467-y

**Published:** 2019-01-09

**Authors:** Junko Matsuzaki, Takemasa Tsuji, Thinle Chodon, Courtney Ryan, Richard C. Koya, Kunle Odunsi

**Affiliations:** 1 Center for Immunotherapy, Department of Immunology, Roswell Park Comprehensive Cancer Center, Buffalo, USA; 2 Center for Immunotherapy, Roswell Park Comprehensive Cancer Center, Buffalo, USA; 3 Center for Immunotherapy, Department of Immunology, Department of Gynecologic Oncology, Roswell Park Comprehensive Cancer Center, Buffalo, USA

**Keywords:** NY-ESO-1, TCR gene-engineering, CD4^+^CD8^+^ double-positive T cells, HLA-A*02:01, Anti-tumor function, Adoptive cell therapy, Ovarian cancer

## Abstract

**Background:**

High-affinity tumor antigen-specific T-cell receptor (TCR) gene is required to engineer potent T cells for therapeutic treatment of cancer patients. However, discovery of suitable therapeutic TCR genes is hampered by the fact that naturally occurring tumor antigen-specific TCRs are generally of low-affinity, and artificial modification of TCRs can mediate cross-reactivity to other antigens expressed in normal tissues. Here, we discovered a naturally occurring T-cell clone which expressed high-affinity HLA-A*02:01 (A*02)-restricted TCR against NY-ESO-1 from a patient who had NY-ESO-1-expressing ovarian tumor.

**Methods:**

A*02-restricted NY-ESO-1-specific T-cell clones were established from peripheral blood of patients who had NY-ESO-1-expressing ovarian tumors. TCR α and β chain genes were retrovirally transduced into polyclonally activated T cells. Phenotype and function of the parental and TCR-transduced T cells were analyzed by flow cytometry, ELISA and cytotoxicity assay. In vivo therapeutic efficacy was investigated in a xenograft model using NOD/SCID/IL-2Rγ-deficient (NSG) mice.

**Results:**

A rare population of NY-ESO-1-specific T cells, which we named 19305DP, expressed cell surface CD4, CD8α, and CD8β but not CD56 and recognized A*02^+^NY-ESO-1^+^ cancer cell lines in a CD4- and CD8-independent manner. 19305DP showed a gene expression profile that is consistent with a mixed profile of CD4^+^ and CD8^+^ single-positive T cells. Both CD4^+^ and CD8^+^ T cells that were retrovirally transduced with 19305DP-derived TCR gene (19305DP-TCR) showed strong reactivity against A*02^+^NY-ESO-1^+^ cancer cells, whereas TCR genes from the conventional A*02-restricted NY-ESO-1-specific CD8^+^ single-positive T-cell clones functioned only in CD8^+^ T cells. Both 19305DP-TCR gene-engineered CD4^+^ and CD8^+^ T cells eliminated A*02^+^NY-ESO-1^+^ tumor xenografts in NSG mice. Finally, based on reactivity against a series of alanine-substituted peptides and a panel of normal human tissue-derived primary cells, 19305DP-TCR was predicted to have no cross-reactivity against any human non-NY-ESO-1 proteins.

**Conclusion:**

Together, our results indicate that the naturally occurring 19305DP-TCR derived from CD4^+^CD8^+^ double-positive αβ T cells**,** is a promising therapeutic TCR gene for effective and safe adoptive T-cell therapy in A*02^+^ patients with NY-ESO-1-expressing tumor.

**Electronic supplementary material:**

The online version of this article (10.1186/s40425-018-0467-y) contains supplementary material, which is available to authorized users.

## Background

Gene-engineering with tumor antigen-specific T-cell receptor (TCR) is a promising strategy for manufacturing therapeutic cell products for adoptive cell therapy (ACT) of cancer patients. Using retroviral or lentiviral vectors, large numbers of autologous tumor antigen-specific T cells can be rapidly manufactured for infusion into patients to provide anti-tumor effector and memory T cells in order to mediate immediate and long-term tumor immune-surveillance. The feasibility, safety, and therapeutic effects of TCR gene-engineered autologous T cells have been demonstrated in clinical trials. Several of these clinical studies have used MHC class I-restricted tumor antigen-specific TCR to manufacture anti-tumor T-cell products [1–3]. It is generally accepted that high-affinity TCR is required to provide efficient tumor-recognizing ability to T cells. This is in part because high-affinity MHC class I-restricted TCR confers tumor-reactivity to CD4^+^ T cells in addition to CD8^+^ T cells by bypassing the requirement of CD8 co-ligation for activation [4–7]. In addition to enhanced anti-tumor effects by CD8^+^ T cells, provision of tumor-reactivity to CD4^+^ T cells by high-affinity TCR may enhance the efficiency of in vivo tumor destruction by cooperative functions of CD8^+^ and CD4^+^ T cells.

Efforts have been made to obtain high-affinity tumor antigen-specific TCR by introducing missense mutations in the complementarity-determining regions in naturally occurring TCR genes [8, 9] and vaccination of HLA-transgenic mice to obtain murine TCRs that were not negatively selected against human antigens [10, 11]. Both methods have generated useful high-affinity TCR genes that have demonstrated safety and therapeutic efficacy in clinical trials. However, in spite of testing these TCR genes for potential cross-reactivity against homologous peptides, some of these artificial TCR genes have caused lethal off-target toxicity in some clinical trials [12, 13], indicating that there are currently no methods to completely predict cross-reactivity of artificially modified TCRs against all proteins expressed in humans. Consequently, a potential solution is to identify tumor-reactive T cells with naturally occurring but exceptionally high-affinity TCR, obviating the need for affinity enhancement. However, such T cells with exceptionally high-affinity are rare because intra-thymic deletion of self-reactive T cells generates a repertoire devoid of high-avidity T-cell clones. Accordingly, self/tumor antigen-specific T-cell repertoire is composed of mainly low-avidity clones in comparison to pathogen-reactive T-cell repertoire, which are generally of high-avidity [14]. Based on recent evidence that clonal deletion prunes, but does not eliminate high-affinity self-reactive T cells [15], we reasoned that identifying these rare T cells could uncover naturally occurring tumor antigen-reactive TCRs of exceptionally high-affinity for potential therapeutic use in ACT.

NY-ESO-1 is one of the most promising targets in immunotherapy of cancers because of high expression in multiple cancer types such as melanoma, synovial sarcoma, ovarian cancer, and lung cancer, whereas its expression in adult normal tissues is restricted to the testis [16, 17]. Although expression of NY-ESO-1 is detectable in thymic epithelial cells [18], which may limit the generation of high-avidity NY-ESO-1-specific T cells, NY-ESO-1 has been considered as one of the most immunogenic tumor antigens and induces strong spontaneous T-cell and antibody responses in patients with NY-ESO-1-expressing tumors [19–21]. In ACT clinical trials in the United States, two high-affinity HLA-A*02:01 (A*02)-restricted NY-ESO-1-specific TCR genes have been utilized to manufacture T-cell products. The first one (named α95:LY or c259) was obtained by mutagenesis [8] and the second one was obtained from A*02 transgenic mice after vaccination [10]. Both TCRs provided A*02-restricted NY-ESO-1-specific reactivity not only to CD8^+^ T cells but also to CD4^+^ T cells, indicating CD8-independent high-affinity recognition. ACT trials using the α95:LY/c259 have demonstrated safety and therapeutic efficacy in melanoma, synovial sarcoma and other tumor types [1, 22, 23]. Clinical trials testing the murine TCR are ongoing (NCT01967823, NCT02774291, and NCT03017131).

In the present study, we unexpectedly discovered a rare population of naturally occurring NY-ESO-1-specific T cells that expressed CD8-independent TCR from peripheral blood of a patient with NY-ESO-1-expressing ovarian cancer. The T-cell clone, which we named 19305DP, expressed both CD4 and CD8 molecules suggesting that this clone escaped thymic negative selection, a process that eliminates self-reactive T cells with high-affinity TCR. 19305DP recognized A*02^+^NY-ESO-1^+^ cancer cells in a CD4- and CD8-independent manner. Both CD4^+^ and CD8^+^ T cells that were engineered with TCR gene from 19305DP showed potent antitumor activity in vitro and in vivo. Finally, no cross-reactivity to human proteins and normal human tissue-derived primary cells was predicted, which supports the safety of the TCR gene to be used in ACT. Our observations suggest that tumor antigen-specific CD4^+^CD8^+^ double-positive T cells could be a useful source for obtaining therapeutic tumor antigen-specific TCR genes for TCR gene-engineering.

## Methods

### NY-ESO-1-specific T cells

NY-ESO-1-specific T-cell clones were established as described previously [24]. Briefly, CD8^+^ and CD4^+^ T cells were sequentially separated from peripheral blood mononuclear cells (PBMC) derived from patients who had spontaneous humoral immune response to NY-ESO-1. To amplify NY-ESO-1-specific T cells, CD4^+^ and CD8^+^ T cells were stimulated with NY-ESO-1_157-170_ peptide (Lausanne Branch of Ludwig Institute for Cancer Research) in separate cultures. To obtain the CD4^+^CD8^+^ double-positive 19305DP T-cell clone, following restimulation with NY-ESO-1 peptide, NY-ESO-1-specific CD4^+^ T cells were isolated by sorting CD40L-expressing cells (FACSAria, BD Biosciences). SK-MEL-37-reactive T cells were then isolated by flow cytometry using IFN-γ secretion assay kit according to manufacturer’s instructions (Miltenyi Biotec). Finally, Vβ8^+^ T cells were magnetically sorted by staining with FITC-conjugated anti-TCR Vβ8 antibody (Clone: 56C5.2, Beckman Courter) followed by incubation with anti-FITC microbeads (Miltenyi Biotec). A*02-restricted NY-ESO-1-specific CD8^+^ T-cell clones (CD8SP1 and CD8SP2) and HLA-Cw*03:04 (Cw*03)-restricted NY-ESO-1-specific CD8^+^ T-cell clone were isolated by cell sorting of A*02/NY-ESO-1_157-165_ or Cw*03/NY-ESO-1_92-100_ tetramer^+^CD8^+^ T cells. CD8SP1 (AL, ESO-CD8 or pt. #20) and Cw*03-restricted CD8^+^ T-cell clone were obtained from ovarian cancer patients who received NY-ESO-1 fowlpox/vaccinia-virus vaccination [25, 26]. CD8SP2 was isolated from the same ovarian cancer patient who had NY-ESO-1-specific CD4^+^CD8^+^ double-positive T cells (19305DP) as described above. NY-ESO-1-specific CD4^+^ T-cell clones (CD4SP1, CD4SP2 and CD4SP3) were isolated by FACSAria using anti-CD40L or IFN-γ secretion assay kit. HLA-DP*04:01 (DP*04)-restricted NY-ESO-1-specific CD4^+^ T-cell clones (CD4SP1: TR-CD4 and CD4SP2: 2C10) were isolated from an ovarian cancer patient who received NY-ESO-1_157-170_ peptide vaccine [24, 27]. HLA-DR*04:04-restricted NY-ESO-1-specific CD4^+^ T-cell clone (CD4SP3: PB-T) was similarly isolated from another NY-ESO-1-seropositive ovarian cancer patient. After each sorting, T cells were expanded with phytohemagglutinin (PHA; Remel) stimulation in the presence of feeder cells (irradiated allogeneic PBMC), IL-2 (Roche Molecular Biochemicals) and IL-7 (R&D systems). All T cells were cultured in RPMI1640 medium supplemented with 10% FBS, penicillin, streptomycin and L-glutamine.

### Cancer cell lines

A375, Mel624.38, Mel888 and Mel938 were kindly provided by Dr. Rosenberg at the National Cancer Institute (NCI). SK-MEL-37, MZ-MEL-19, NW-MEL-38 and SK-MEL-29 were obtained from New York branch of Ludwig Institute for Cancer Research. MZ-MEL-9 and MZ-MEL-12 were kindly provided by Dr. Jäger (Krankenhaus Nordwest, Frankfurt Germany). Epithelial ovarian cancer cell lines, 19305EOC and 18637EOC, were established from single cell suspension of ovarian tumor specimens at Roswell Park Comprehensive Cancer Center. All cancer cells were cultured in RPMI1640 medium supplemented with 10% FBS, penicillin, streptomycin and L-glutamine.

### Nanostring analysis

19305DP, NY-ESO-1-specific CD8 single positive T-cell clones (CD8SP) and NY-ESO-1-specific CD4^+^ T-cell clones (CD4SP) (0.5 × 10^6^) were untreated or stimulated with immobilized anti-CD3 monoclonal antibody (clone OKT3, 5 μg/ml, eBioscience) for 2 h. The cells were harvested and washed with PBS, and then the cell pellets were frozen. RNA extraction and gene expression analyses using Nanostring PanCancer Immune Panel on nCounter Analysis System version 2.6 were performed at the Genomics Shared Resource at Roswell Park Comprehensive Cancer Center. The heat map was generated by the Heatmapper tool (http://bar.utoronto.ca/ntools/cgi-bin/ntools_heatmapper.cgi) or the nSolver Analysis Software version 4.0.

### Retroviral transduction of TCR gene

Full-length TCR α and β chain genes were cloned by 5’-RACE PCR method and sequenced as previously described [28]. TCR β chain gene was genetically fused to α chain gene via T2A site and cloned into a murine stem cell virus-derived retroviral vector. Retroviral vector production and transduction were performed as previously described [26]. Transduction efficiency was determined by staining with A*02/NY-ESO-1_157-165_ (SLLMWITQC) tetramer (iTAg MHC tetramer, MBL), anti-human TCR Vβ8 mAb (clone JR2, BioLegend) for 19305DP-TCR, and anti-human TCR Vβ3 mAb (clone CH92, Beckman Coulter) for CD8SP (CD8SP1) or control HLA-DR*01:01 (DR*01)-restricted NY-ESO-1-specific TCR [29]. In some experiments, CD4^+^ or CD8^+^ T cells were depleted from PBMC by anti-biotin magnetic beads (Dynabeads, Life Technologies) following staining with biotin-conjugated anti-CD4 (clone OKT4, BioLegend) or anti-CD8 (clone HIT8a, BioLegend) mAb prior to activation with PHA. For in vivo experiments, T cells were harvested 20–22 h after the second infection.

### In vitro cytotoxicity assay

In vitro cytotoxicity assay was performed by a calcein-AM release assay. Mel624.38, A375 or MZ-MEL-12 were labeled with 5 μM calcein-AM in the presence of 0.05% F-127 for 30 min, washed, and cocultured with T cells for 4 h. The supernatant was harvested and fluorescence was read using Synergy HT microplate reader (BioTek) with 485/20 excitation and 528/20 emission filters. To determine spontaneous and maximum release, target cells were cultured in culture medium in the absence and presence of 2% Triton-X, respectively. Cytotoxicity was calculated using the following formula: % cytotoxicity = 100 × [(experimental release – spontaneous release)/(maximum release – spontaneous release)].

### In vivo xenograft mouse model

A375 cells (1 × 10^6^) were subcutaneously inoculated in NOD/SCID/IL-2Rγ-deficient (NSG) mice (Jackson laboratories). At day 11, 2.5 × 10^5^ T cells were intravenously injected. Starting on the day of T-cell transfer, recombinant human IL-2 (5 × 10^4^ U; PeproTech) was intraperitoneally injected for 3 days. Tumor size was measured by calipers every 2–3 days following injection. Tumor volume was calculated by the following formula; 0.5 × (length × width × width). Mice were euthanized when the tumor volume reached 2000 mm^3^ per the institutional regulation guideline.

### Phenotypic and functional analysis

Phenotype of T cells was analyzed by flow cytometry using antibodies for cell surface molecules [CD3 (clone: HIT3a, BioLegend), CD4 (clone: OKT4, BioLegend), CD8α (clone: RPA-T8, BioLegend), CD8β (clone: SIDI8BEE, eBioscience), CD56 (clone: HCD56, BioLegend), TCRαβ (clone: T10B9, BD Biosciences), TCRγδ (clone: B1, BioLegend), and Cw*03/NY-ESO-1_92-100_ (LAMPFATPM) tetramer (Lausanne branch of Ludwig Institute for Cancer Research)]. Intracellular IFN-γ (clone B27, BD Biosciences) staining was performed using Fix & Perm Medium B (Life Technologies) following 6 h stimulation with target cells in the presence of 5 μg/ml monensin (Sigma). Epstein-Barr virus-transformed B (EBV-B) cells were pulsed overnight with NY-ESO-1_157-170_ peptide (10 μM), NY-ESO-1_157-165_ peptide (10 μM, Lausanne branch of Ludwig Institute for Cancer Research) or NY-ESO-1 protein (10 μg/ml, New York branch of Ludwig Institute for Cancer Research) [26]. In vitro transcribed NY-ESO-1 mRNA was electroporated in EBV-B cells as described previously [30]. In some experiments, cancer cells were incubated for 30 min with antibodies for HLA-A,B,C (clone W6/32, 20 μg/ml, BioLegend) or HLA-DR,DP,DQ (clone Tu39, 10 μg/ml, BioLegend), or T cells were incubated with antibodies for CD4 (clone RPA-T4, 10 μg/ml, BioLegend) or CD8 (clone SK1, 10 μg/ml, BioLegend) prior to mixing. Propidium iodide (PI) and anti-annexin V staining on cancer cells after 24 h coculture with T cells was performed using the FITC Annexin V Apoptosis Detection Kit I (BD Biosciences). For testing IFN-γ levels in culture supernatant, TCR-transduced whole PBMC, CD4^+^ T cells or CD8^+^ T cells (5 × 10^4^) were cultured with A375 (2.5 × 10^4^) in a 96-well round-bottom culture plate. The culture supernatant was collected day 1 to day 4 after the culture and stored at − 20 °C until measurement of IFN-γ by ELISA according to manufacturer’s instruction (eBioscience). For testing avidity of TCR-transduced T cells against NY-ESO-1_157-165_ wild-type and alanine substituted peptides (Bio-Synthesis Inc), the titrated peptides from 10 μM to 100 pM were pulsed on A*02^+^ EBV-B cells. Percentage of IFN-γ-producing CD8^+^ and CD4^+^ T cells was determined by intracellular cytokine staining.

### T-cell reactivity against normal human tissue-derived primary cells

A panel of normal human tissue-derived primary cells representing various organs in the body was obtained from ScienCell Research Laboratories. All primary cells were cultured according to manufacturer’s instructions. A*02 typing was performed using PCR-SSP method [31] at the Immune Analysis Shared Resource at Roswell Park Comprehensive Cancer Center. For the human primary cells that were not naturally A*02^+^, A*02-expressing lentiviral vector was utilized to induce the expression. 19305DP-TCR-transduced T cells (2 × 10^5^) were cocultured with normal primary cells (2 × 10^4^ cells plated the day before) for 24 h and IFN-γ levels in the culture supernatants were determined by ELISA (eBioscience). Melanoma cell lines with or without A*02 and/or NY-ESO-1 expression were used as assay controls. To provide additional positive controls, some types of normal primary cells were pulsed with NY-ESO-1_157-165_ peptide before coculture.

### Statistical analyses

Data are shown as means and standard deviations. Survival curve was plotted and analyzed using prism 7.03 software (GraphPad Software). *P* values of less than 0.05 were considered statistically significant by unpaired Student’s *t*-test.

## Results

### Generation of a tumor antigen-specific CD4^+^CD8^+^ double-positive T-cell clone

**Fig. 1:**
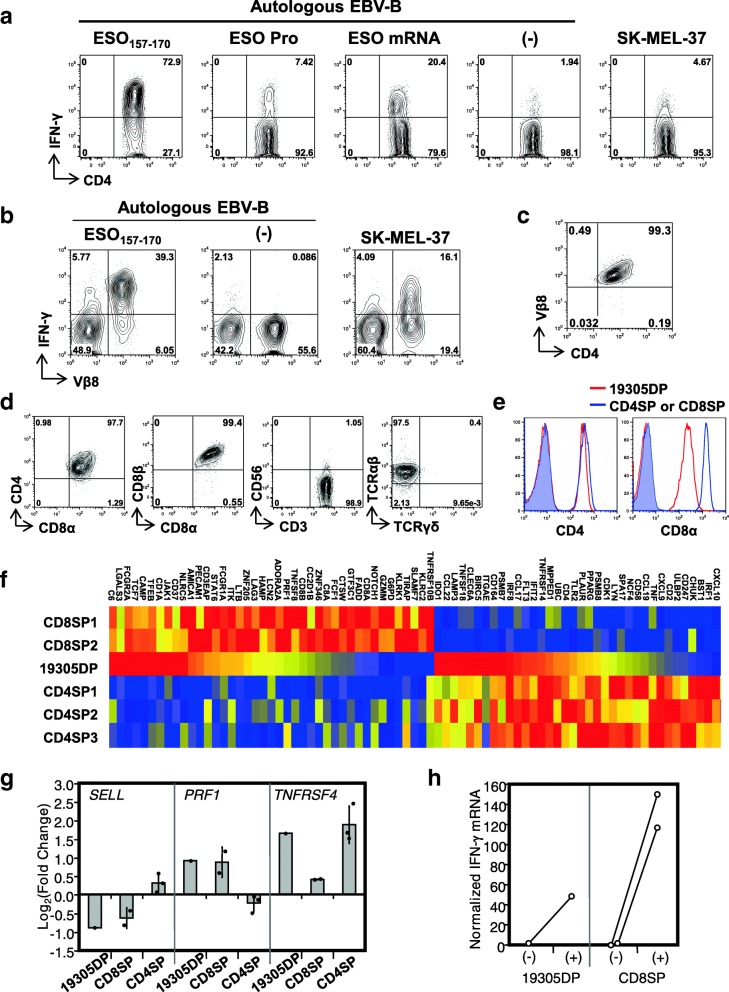
Characterization of NY-ESO-1-specific CD4^+^CD8^+^ T cells. **a** IFN-γ production by NY-ESO-1-specific CD4^+^ T-cell line was determined by intracellular cytokine staining following stimulation with NY-ESO-1_157-170_ peptide-pulsed, protein-pulsed, mRNA-electroporated or untreated (−) autologous EBV-transformed B cells (EBV-B), or SK-MEL-37. **b** IFN-γ production by SK-MEL-37-reactive CD4^+^ T-cell line was determined by flow cytometry. **c** Purification of CD4^+^Vβ8^+^ T cells was confirmed by flow cytometry. **d** Expression of cell surface molecules on 19305DP was determined by flow cytometry. **e** Expression of CD4 and CD8α on 19305DP was compared to those in CD4SP (left) and CD8SP (right) by flow cytometry. Shaded histograms indicate staining by isotype controls (mouse IgG2b for CD4 and mouse IgG1 for CD8α). **f** Gene expression in T-cell clones was investigated by Nanostring system. Expression of genes that were differently expressed in NY-ESO-1-specific CD8^+^ T-cell clones (CD8SP1 and 2) and NY-ESO-1-specific CD4^+^ T-cell clones (CD4SP1–3) in 19305DP without stimulation is shown as a heat map (red, yellow, and blue colors indicates strong, intermediate, and low expression, respectively). **g** Changes in mRNA expression following TCR stimulation was determined by Nanostring data. Columns and error bars for CD8SP and CD4SP indicate the means and the standard deviation. **h** Changes in IFN-γ mRNA levels with or without anti-CD3 stimulation in Nanostring data

In previous studies, we reported that a subset of NY-ESO-1-specific CD4^+^ T cells directly recognizes NY-ESO-1-expressing cancer targets in a MHC class II-restricted manner (tumor-recognizing CD4^+^ T cells or TR-CD4 cells) [24, 29]. In one of our experiments testing TR-CD4-cell response in a DP*04^+^ patient with exceptionally high-titer spontaneous anti-NY-ESO-1 serum antibody (reciprocal titers: > 10,000,000), we established a DP*04-binding NY-ESO-1_157-170_ peptide-specific CD4^+^ T-cell line and tested direct tumor recognition. A small but significant fraction of the CD4^+^ T-cell line recognized a NY-ESO-1^+^DP*04^+^ SK-MEL-37 melanoma cell line, indicating the presence of TR-CD4 cells (Fig. 1a). Consistent with our previous observations for TR-CD4 cells [24, 29], the CD4^+^ T cells strongly recognized cytoplasmic NY-ESO-1 expressed by electroporation with in vitro-transcribed mRNA. However, in contrast to TR-CD4 cells, the cells poorly recognized target cells pulsed with recombinant NY-ESO-1 protein.

To further investigate direct tumor recognition by this NY-ESO-1_157-170_-specific CD4^+^ T cells, SK-MEL-37-recognizing CD4^+^ T cells were isolated by flow cytometric sorting and were expanded. Through low-resolution TCR Vβ spectratyping using a panel of Vβ subtype-specific antibodies, we found that SK-MEL-37-recognizing CD4^+^ T cells express TCR Vβ8 (Fig. 1b). To obtain monoclonal population, TCR Vβ8^+^ T cells were further purified for characterization of their function and phenotype (Fig. 1c). We found that this T-cell clone co-expressed cell surface CD8α and CD8β in addition to CD4 and was positive for TCR αβ chains but not γδ chains (Fig. 1d). The CD4^+^CD8^+^ double-positive T-cell clone, which we named 19305DP, was negative for cell surface CD56, excluding the possibility that they were natural killer T cells. Expression of CD8α/CD8β heterodimers also excludes the possibility that 19305DP is a subset of intraepithelial T cells that expresses CD8αα homodimers in addition to CD4 [32]. When compared with a CD4^+^ single-positive T-cell clone (CD4SP), 19305DP expressed similar level of cell surface CD4, whereas its cell surface CD8α and CD8β expression level was significantly lower than a CD8^+^ single-positive T-cell clone (CD8SP) (Fig. 1e and data not shown).

### Characterization of 19305DP

To gain insight into the ontogeny of 19305DP, we compared the gene expression pattern of 19305DP with those of three NY-ESO-1-specific CD4SP clones or two NY-ESO-1-specific CD8SP clones, with or without TCR stimulation using the Nanostring nCounter PanCancer Immune panel. Clustering analysis suggested that 19305DP had distinguishable gene expression profile from those of CD4SP (CD4SP1–3) or CD8SP (CD8SP1 and 2) clones (Additional file 1). Similar expression of CD4 and decreased expression of CD8α as compared to CD4SP and CD8SP clones, respectively, were confirmed at mRNA expression levels (Fig. 1f). In addition, CD8β mRNA level in 19305DP was about half of CD8SP clones. At baseline without stimulation, 19305DP expressed a fraction of genes that are preferentially expressed in CD4SP clones such as *IDO1*, *CCL22*, and *CCL17*. In addition, *PECAM1*, *AMICA1*, *LAG3*, and *LTB* that were significantly overexpressed in CD8SP clones compared to CD4SP clones were expressed in unstimulated 19305DP (Fig. 1f). After stimulation, 19305DP upregulated *TNFRSF4* (OX40; CD134) similarly to CD4SP clones whereas the expression of *PRF1* (perforin 1) and *SELL* (L-selectin; CD62L) was changed similarly to CD8SP clones (Fig. 1g). This gene expression profile supports that 19305DP is a distinct T-cell subset expressing characteristic genes for both CD4^+^ and CD8^+^ T cells.

**Fig. 2:**
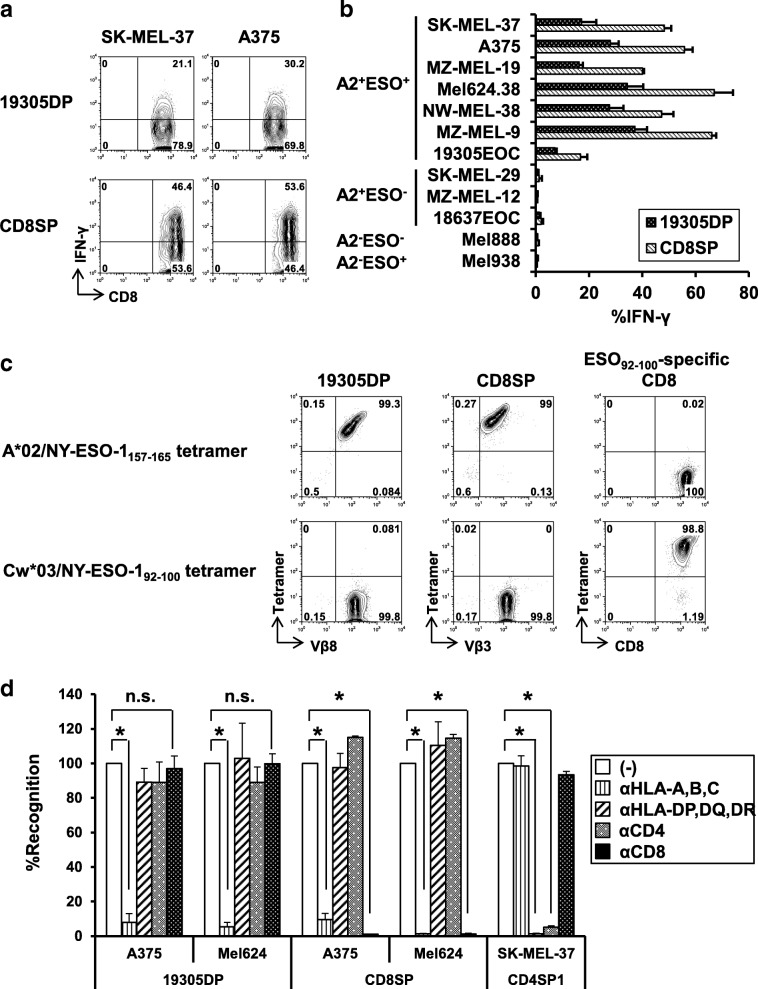
Comparison of cancer-cell recognition by A*02-restricted NY-ESO-1-specific CD4^+^CD8^+^ double-positive 19305DP and CD8^+^ single-positive CD8SP. **a** IFN-γ production from 19305DP and CD8SP (CD8SP1) against A*02^+^NY-ESO-1^+^ melanoma cell lines (SK-MEL-37 and A375) was determined by intracellular cytokine staining. **b** The reactivity of 19305DP and CD8SP against a panel of cancer cell lines with different A*02 (A2) and NY-ESO-1 (ESO) expression was tested by intracellular IFN-γ staining. **c** A*02/NY-ESO-1_157-165_ tetramer binding and TCR Vβ expression was determined by flow cytometry. Cw*03-restricted NY-ESO-1-specific CD8^+^ T-cell clone and Cw*03/NY-ESO-1_92-100_ tetramer were used as controls to demonstrate specific tetramer binding. **d** The effect of blocking antibodies for MHC class I (αHLA-A,B,C), MHC class II (αHLA-DP,DQ,DR), CD4 (αCD4) or CD8 (αCD8) on recognition of the indicated melanoma cell lines was investigated by intracellular IFN-γ staining. The data was represented as % recognition as compared to the recognition without antibodies (−). *p < 0.05 compared without antibody treatment

By testing reactivity against a panel of NY-ESO-1-expressing, NY-ESO-1-non-expressing, A*02^+^, and non-A*02^+^ cancer cell lines together with control A*02-restricted NY-ESO-1-specific CD8SP1 clone, direct tumor recognition by 19305DP was found to be NY-ESO-1-specific and A*02-restricted (Fig. 2a and b). Among cell lines tested, surface MHC class II-expressing (SK-MEL-37, A375 and MZ-MEL-19) and non-expressing cell lines (MEL624.38, NW-MEL-38 and MZ-MEL-9) were similarly recognized by 19305DP, indicating that co-ligation of CD4 molecules did not significantly contribute to the recognition in contrast to observations for HLA-A2-restricted H-Y-specific CD4^+^ T cells or MHC class I-restricted alloreactive CD4^+^ T cells [33, 34]. 19305DP recognized autologous ovarian cancer cell line (19305EOC) which expressed NY-ESO-1 and A*02 at lower levels than other A*02^+^ melanoma cell lines (Additional file 2). IFN-γ production from 19305DP was consistently weaker than the conventional NY-ESO-1-specific CD8SP, which was consistent with the observation that IFN-γ mRNA level after anti-CD3 antibody stimulation was less than half of those of CD8SP clones (Fig. 1h). Because 19305DP recognition of cancer cells was restricted by A*02, tetramer binding of 19305DP to A*02/NY-ESO-1_157-165_ tetramer was examined (Fig. 2c). Similar to the A*02-restricted NY-ESO-1-specific CD8SP clone which expressed TCR-Vβ3, TCR-Vβ8^+^ 19305DP was stained by the A*02/NY-ESO-1_157-165_ tetramer but not by the control Cw*03/NY-ESO-1_92-100_ tetramer.

Next, we assessed whether co-ligation of CD8 or CD4 molecules on 19305DP to MHC class I or II, respectively, contributed to T-cell reactivity using anti-CD8 and anti-CD4 blocking antibodies and in addition, using anti-MHC class I and class II blocking antibodies. As expected, recognition of A*02^+^NY-ESO-1^+^ melanoma cells by both 19305DP and CD8SP was abrogated by blocking MHC class I (Fig. 2d). In sharp contrast to complete inhibitory effect of anti-CD8 mAb on recognition by CD8SP, the same antibody (10 μg/ml) did not inhibit the recognition by 19305DP, indicating that TCR in 19305DP transduces activation signals in the absence of CD8 co-ligation. In addition, consistent with efficient recognition of MHC class II-negative cancer cell lines (Fig. 2b), MHC class II and CD4 co-ligation was not involved in the TCR activation, as anti-MHC class II and anti-CD4 blocking antibody showed no effects on recognition by 19305DP whereas these antibodies significantly inhibited SK-MEL-37 recognition by MHC class II-restricted TR-CD4 (CD4SP1) (Fig. 2d).

### Generation of TCR-expressing retroviral vectors and comparative analysis with affinity matured TCR

**Fig. 3:**
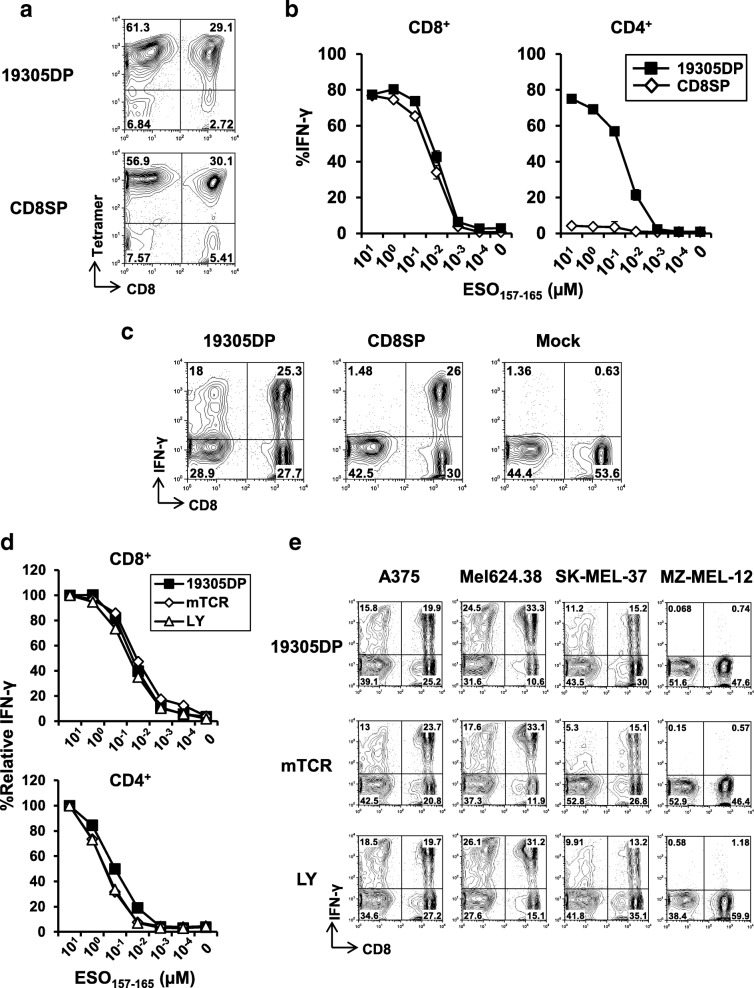
TCR avidity and tumor recognizing capacity of TCR gene-transduced T cells. **a** TCR transduction efficiency on normal donor PBMC was determined by tetramer staining. **b** TCR avidity of TCR-transduced CD8^+^ and CD4^+^ T cells against NY-ESO-1_157-165_ peptide-pulsed A*02^+^ EBV-B cells was determined by intracellular IFN-γ staining. The data indicates means ± SD from duplicate experiment. **c** Representative flow cytometry plots of IFN-γ production on CD8^+^ and CD8^−^ (CD4^+^) T cells against A375 is shown. **d** TCR avidity of 19305DP-TCR, murine TCR (mTCR) or affinity-enhanced TCR (LY)-transduced CD8^+^ and CD4^+^ T cells was determined against NY-ESO-1_157-165_ peptide-pulsed A*02^+^ EBV-B cells by intracellular IFN-γ staining. Data are represented as % relative IFN-γ production at different peptide concentration as compared to % IFN-γ production at 10 μM. **e** Recognition of the indicated melanoma cell lines by 19305DP-TCR-transduced T cells was compared to T cells transduced with LY or mTCR recognizing the same NY-ESO-1-epitope region

Because of the minimal requirement for CD8 co-ligation in recognition of cancer targets by 19305DP, we reasoned that this clone expressed high-affinity CD8-independent TCR [7, 35]. Therefore, we investigated whether naturally occurring TCR from 19305DP without affinity enhancement could transfer high-avidity recognition of cancer cells to donor CD4^+^ T cells in addition to CD8^+^ T cells by retroviral TCR gene-engineering. Full-length TCR α and β chain-coding genes were cloned from 19305DP. Only a single pair of TCR α and β chain genes was obtained for 19305DP, confirming that 19305DP was T-cell clone. There was no mutation in their TCR α and β chain variable, joining and constant regions by the IMGT/V-QUEST platform (Additional file 3) [36]. Full DNA and amino acid sequences for 19305DP-TCR are also available in the published patent application: WO2017120428. The schematic representation of the TCR-expressing plasmid vector is shown in Additional file 4A. TCR gene from CD8SP1 was used as a control CD8-dependent TCR recognizing the same NY-ESO-1 epitope region. Using our optimized protocol [26], transduction efficiency was routinely above 85%, as determined by tetramer and TCR Vβ staining by flow-cytometry (Fig. 3a and Additional file 4B). Both CD4^+^ and CD8^+^ T cells were similarly transduced to express Vβ of transgenic TCR for both 19305DP and CD8SP (Additional file 5B).

TCR avidity of 19305DP-TCR- and CD8SP-TCR-transduced CD8^+^ and CD4^+^ T cells was determined against peptide-pulsed A*02^+^ EBV-B cell line by intracellular staining (Fig. 3b). TCR avidity to recognize NY-ESO-1_157-165_ peptide was not different between 19305DP-TCR and CD8SP-TCR when they were expressed on CD8^+^ T cells. 19305DP-TCR-transduced CD4^+^ T cells showed similar dose-dependence with 19305DP-TCR-transduced CD8^+^ T cells to recognize peptide-pulsed targets. In contrast, CD8SP-TCR-transduced CD4^+^ T cells showed negligible reactivity to the peptide. Recognition of A*02^+^NY-ESO-1^+^ cancer cell lines by TCR gene-transduced T cells was also investigated by intracellular IFN-γ staining. Both 19305DP-TCR and CD8SP-TCR induced strong IFN-γ production from CD8^+^ T cells (Fig. 3c and Additional file 5). Consistent with CD8-independent and -dependent target cell recognition by 19305DP and CD8SP, respectively (Fig. 2d), 19305DP-TCR but not CD8SP-TCR provided efficient tumor-recognizing ability to CD4^+^ T cells. This observation was confirmed with CD8SP2-TCR gene-engineered T cells (data not shown). Furthermore, consistent with results using parental T-cell clones (Fig. 2d), reactivity of 19305DP-TCR gene-transduced CD8^+^ and CD4^+^ T cells was not blocked by anti-CD8, anti-CD4 nor anti-MHC class II antibodies in contrast to strong inhibition by anti-MHC class I antibody (Additional file 6 and data not shown).

We then compared the reactivity of 19305DP-TCR-transduced T cells with T cells retrovirally transduced with the affinity enhanced NY-ESO-1-specific TCRs: α95:LY/c259 (LY) and murine TCR (mTCR) that have been used in previous and ongoing clinical trials (LY: NCT01567891, NCT01350401, NCT01892293, NCT03391778, NCT01352286, NCT02588612, NCT03168438, NCT01343043 and NCT02992743; mTCR: NCT01967823, NCT02774291 and NCT03017131) [22, 23]. LY and mTCR TCR genes were kindly provided by Dr. Rosenberg at the NCI. Functional avidity of 19305DP-TCR-transduced CD8^+^ and CD4^+^ T cells against NY-ESO-1_157-165_ peptide was similar to those of LY-TCR or mTCR-transduced T cells (Fig. 3d). In addition, as shown in Fig. 3e, recognition of A*02^+^NY-ESO-1^+^ cancer cell lines by 19305DP-TCR-transduced CD4^+^ and CD8^+^ T cells was comparable to those engineered with LY and mTCR. Importantly, whereas we observed that CD8^+^ T cells transduced with LY or mTCR produced IFN-γ against some cancer cell lines that lack NY-ESO-1 or A*02 expression, off-target reactivity was not observed from 19305DP-TCR-transduced cells (Additional file 7). Because off-target reactivity by LY and mTCR has not been previously reported [8, 10, 23], a potential explanation is that our engineered T cells were in vitro differentiated to IFN-γ-producing type 1 T cells (Th1 and Tc1). Therefore, it is possible that our assays sensitively detected response against weak stimulation. These results indicate that even without affinity enhancement, 19305DP-TCR provides tumor-recognizing ability to both donor CD8^+^ and CD4^+^ T cells and has sufficient affinity which is comparable to affinity-enhanced or murine TCRs, and superior specificity, both of which are critical attributes for an ideal TCR to be utilized for clinical trials.

### Anti-tumor activity of TCR gene-engineered T cells

CD8^+^ and CD4^+^ T cells play distinct roles in anti-tumor immune responses. Generally, CD8^+^ T cells are considered as effector cells to destroy cancer cells, whereas the roles of anti-tumor CD4^+^ T cells are to provide CD4-help to other immune effector cells such as CD8^+^ T cells through cytokine production and CD40-CD40L ligation. In order to separately investigate anti-tumor functions of CD8^+^ and CD4^+^ T cells transduced with 19305DP-TCR, we polyclonally activated and retrovirally infected PBMC depleted of CD4^+^ or CD8^+^ cells to obtain TCR gene-transduced CD8^+^ or CD4^+^ T cells, respectively. Using this protocol, purity of CD4^+^ and CD8^+^ T cells were more than 90% and transduction efficiency was over 85% (Additional file 8). In addition to 19305DP- and CD8SP-TCRs, irrelevant NY-ESO-1-specific DR*01-restricted TCR, which did not recognize A375 and Mel624.38 was used as a control TCR [29].

**Fig. 4:**
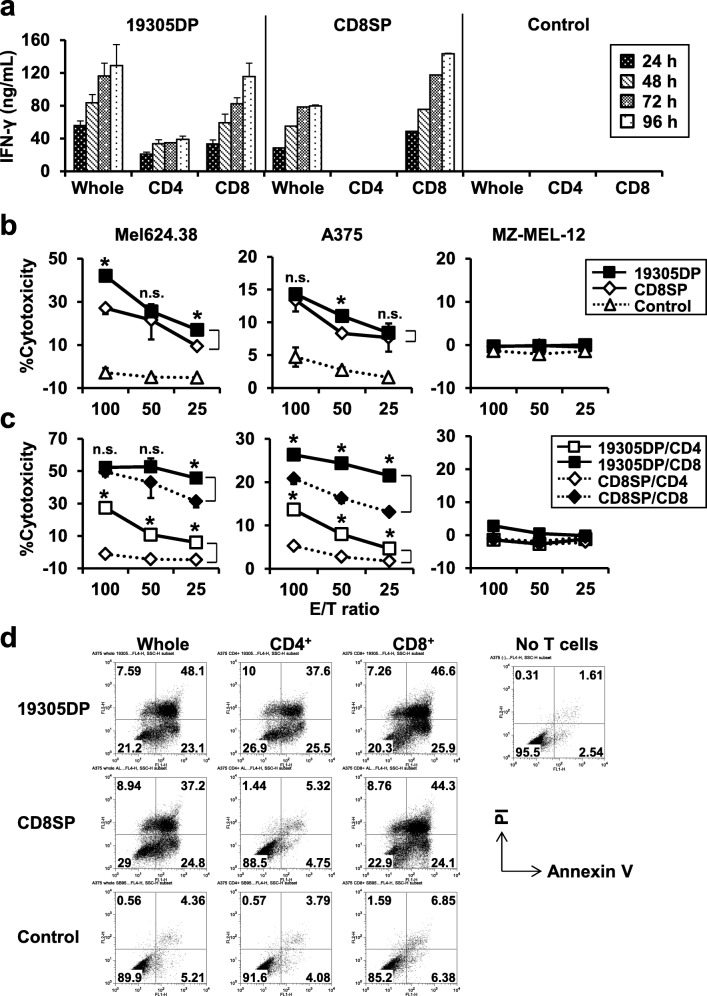
IFN-γ production and in vitro cytotoxicity of TCR-transduced T cells against cancer cells. **a** Whole PBMC, CD4^+^ or CD8^+^ T cells that were transduced with 19305DP-TCR, CD8SP-TCR, or control irrelevant TCR gene were cocultured with A375 and the culture supernatant was harvested at day 1 - day 4. IFN-γ levels in the supernatant were measured by ELISA. **b** Whole PBMC were transduced with 19305DP-TCR, CD8SP-TCR or control-TCR and tested for cytotoxicity against the indicated melanoma cell lines in a calcein-AM release assay. *p < 0.05 compared with CD8SP. **c** Cytotoxic activity of isolated CD4^+^ or CD8^+^ T cells transduced with 19305DP-TCR or CD8SP-TCR against the same panel of melanoma cell lines in (**b**) was tested. *p < 0.05 compared with CD8SP-CD4 or CD8SP-CD8, respectively. **d** A375 and TCR-transduced T cells were cocultured at 1:2 ratio in a 24-well culture plate for 24 h. The cells were harvested with 0.25% trypsin treatment and stained with anti-CD3 antibody followed by FITC annexin V apoptosis detection kit. Annexin V and PI expression on cancer cells gated by FCS/SSC profile and negative CD3 expression were plotted

As expected, separated CD4^+^ and CD8^+^ T cells were fully functional to recognize cancer cells to produce IFN-γ when they were transduced by 19305DP-TCR, whereas only CD8SP-TCR-transduced CD8^+^ T cells but not CD4^+^ T cells were reactive against cancer cells (Fig. 4a). Production of other effector cytokine and cytotoxic molecules from TCR gene-engineered CD4^+^ and CD8^+^ T cells against cancer cells is shown in Additional file 9. 19305DP-TCR-transduced CD4^+^ T cells produced significantly higher amount of TNF-α and IL-2 than CD8^+^ T cells. It is of note that CD8SP-TCR-transduced CD8^+^ T cells produced higher levels of IFN-γ, TNF-α and IL-2 than 19305DP-TCR transduced CD8^+^ T cells, but comparable levels of perforin and granzyme B. Especially, IL-2 was barely detectable in cultures of 19305DP-TCR-transduced CD8^+^ T cells, whereas they were significantly produced from 19305DP-TCR-transduced CD4^+^ T cells. These results potentially indicate a negative regulation of cytokine production or high consumption of cytokines on CD8^+^ T cells following CD8-independent TCR-signaling.

Cytotoxicity of TCR-transduced unseparated T cells, and isolated CD4^+^ and CD8^+^ T cells was investigated by a 4-h calcein-AM release assay (Fig. 4b and c). Both 19305DP-TCR and CD8SP-TCR-transduced T cells showed specific cytotoxic activity against A*02^+^NY-ESO-1^+^ Mel624.38 and A375 but not A*02^+^NY-ESO-1^−^ MZ-MEL-12. Unseparated 19305DP-TCR-transduced PBMCs containing both CD4^+^ and CD8^+^ T cells showed stronger cytotoxicity than CD8SP-TCR-transduced PBMCs (Fig. 4b). As shown in Fig. 4c, 19305DP-TCR-transduced CD8^+^ T cells showed stronger cytotoxicity than CD8SP-TCR-transduced CD8^+^ T cells. In addition, 19305DP-TCR-transduced CD4^+^ T cells showed significant but weaker cytotoxicity compared with CD8^+^ T cells. Therefore, increased cytotoxicity by 19305DP-TCR-transduced unseparated PBMCs is explained by both increased reactivity of CD8^+^ T cells and cytotoxicity of CD4^+^ T cells. It is also possible that CD4^+^ T cells provide CD4-help to enhance cytotoxicity by CD8^+^ T cells. 19305DP-TCR-transduced T cells also induced more annexin V^+^ PI^+^ late apoptotic/dead cells compared with CD8SP-TCR-transduced T cells after 24-h culture at 2:1 E/T ratio (Fig. 4d). Although our 19305DP-TCR-transduced CD4^+^ T cells showed moderate cytotoxicity, it is possible that production of anti-tumor effector cytokines such as IFN-γ and TNF-α from CD4^+^ T cells may damage cancer cells [26]. Indeed, 19305DP-TCR-transduced CD4^+^ T cells significantly induced expression of the apoptotic marker annexin V and PI on cell surface of cancer cells after 24-h co-culture (Fig. 4d).

### In vivo therapeutic effect of TCR gene-engineered T cells

Next, we investigated the in vivo therapeutic efficacy of adoptively transferred TCR gene-engineered T cells in a human tumor xenograft model. NSG mice were subcutaneously inoculated with an A*02^+^NY-ESO-1^+^ human melanoma cell line, A375. Eleven days after inoculation when large tumors (> 7 × 4 mm in diameter) were established, TCR gene-engineered T cells were intravenously injected.

**Fig. 5:**
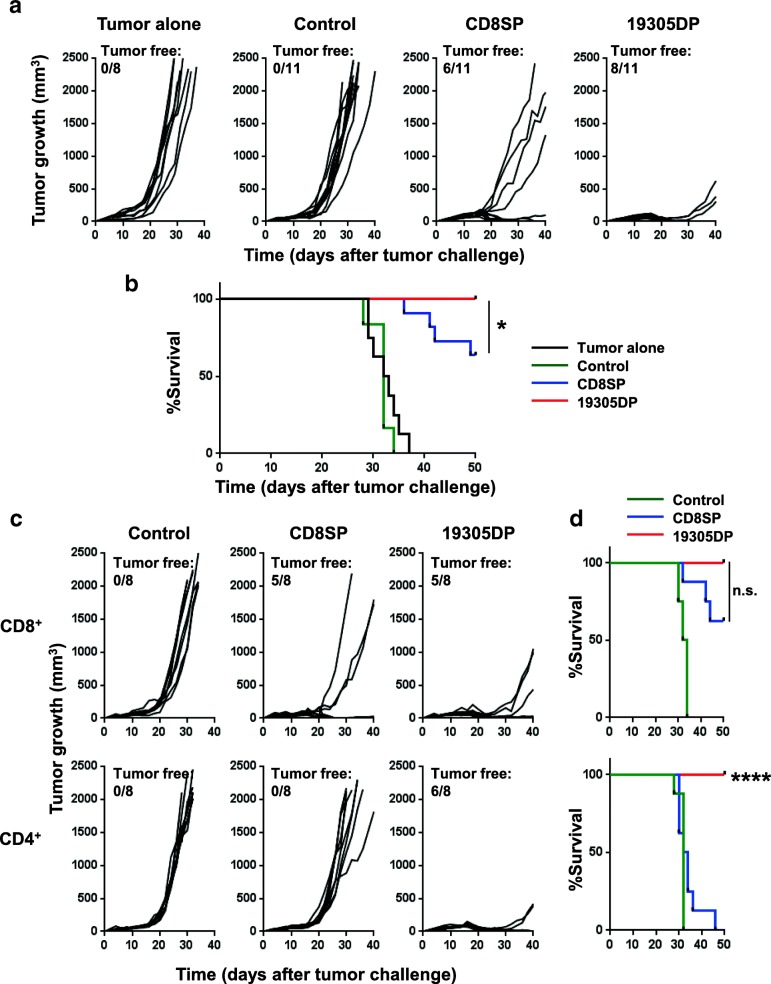
In vivo anti-tumor activity by 19305DP-TCR or CD8SP-TCR-transduced T cells. A375 (1 × 10^6^ cells) was subcutaneously inoculated in NSG mice at day 0. At day 11, 2.5 × 10^5^ TCR-transduced T cells were intravenously transferred. Tumor volume was calculated from tumor diameter measured every 2–3 days. **a** Tumor growth in mice that received whole PBMC-expressing 19305DP-TCR (*n* = 11), CD8SP-TCR (*n* = 11) or NY-ESO-1-specific DR*01-restricted control-TCR-transduced T cells (*n* = 11) are shown. Tumor alone group received no T cells or IL-2 (*n* = 8). **b** Survival of mice in (**a**) is plotted. **c** Tumor growth in mice that received isolated CD8^+^ or CD4^+^ T cells expressing 19305DP-TCR, CD8SP-TCR or control-TCR is shown (*n* = 8 per group). **d** Survival of mice in (**c**) is shown. Mice were considered to reach endpoint when tumor volume exceeded 2000 mm^3^. *p < 0.05, ****p < 0.0001 compared with CD8SP

First, we compared therapeutic effects with 2.5 × 10^5^ unseparated T cells that constituted of 65–70% CD4^+^ and 20–25% CD8^+^ T cells as determined by flow cytometry. As shown in Fig. 5a, CD8SP-TCR-transduced T cells showed significantly delayed tumor growth as compared to the control TCR-transduced T cells, and complete tumor regression was observed in 6/11 mice. As expected, 19305DP-TCR-transduced T cells eliminated tumor more efficiently with complete tumor regression in 8/11 mice and significantly improved survival compared to the CD8SP-TCR (Fig. 5b).

To understand the mechanism behind the more efficient therapeutic effect of 19305DP-TCR compared to CD8SP-TCR, and to determine in vivo therapeutic effects of MHC class I-restricted CD4^+^ T cells, we adoptively transferred separated CD4^+^ or CD8^+^ TCR gene-engineered T-cell products (2.5 × 10^5^). As shown in Fig. 5c, 19305DP-TCR-transduced CD8^+^ T cell alone mediated a slightly better tumor control as compared to CD8SP-TCR-transduced CD8^+^ T cells. As expected from the lack of reactivity to this cancer cell line in vitro (Figs. 3 and 4), CD8SP-TCR-transduced CD4^+^ T cells showed negligible inhibition of tumor growth in vivo. Interestingly, in contrast to the moderate in vitro cytotoxicity compared with CD8^+^ T cells, 19305DP-TCR-transduced CD4^+^ T cell alone showed efficient inhibition of tumor growth to a similar degree as that observed with 19305DP-TCR-transduced CD8^+^ T cells in vivo. Taken together, our results indicated that both CD4^+^ and CD8^+^ T cells potently inhibit in vivo tumor growth when they are engineered by high-affinity 19305DP-TCR.

### Absence of cross-reactivity by 19305DP-TCR to self-antigens in normal cells

Expression of both CD4 and CD8 molecules on 19305DP and CD8-independent recognition of NY-ESO-1-expressing cancer cells raise the possibility that 19305DP T-cell clone escaped from thymic negative selection which removes high-affinity T cells reactive against self-proteins including tissue-restricted and tumor-associated antigens [18]. Although previous clinical trials have demonstrated that immunotherapies including ACT targeting NY-ESO-1 is safe in patients, it is critical to confirm that 19305DP-TCR gene-engineered T cells are reactive only to NY-ESO-1 and not cross-reactive to any human proteins that are expressed in the body. To determine antigen-specificity of 19305DP-TCR, we first determined amino acid residues that are critical to interact with TCR and A*02 using alanine-substituted peptides. As a control TCR, we also tested CD8SP-TCR which is considered to have been selected by thymic negative selection because it shows the CD8-dependent recognition. We synthesized a series of NY-ESO-1_157-165_ peptides in which each amino acid residue in A*02-binding epitope (NY-ESO-1_157-165_: SLLMWITQC) was replaced by the alanine residue.

**Fig. 6:**
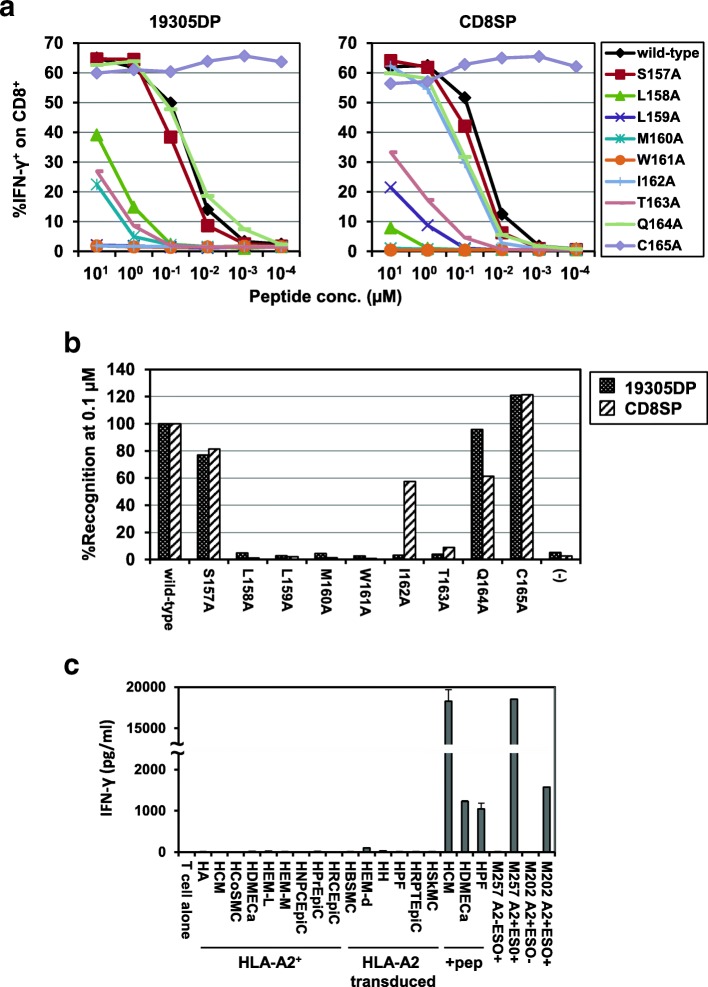
Specificity of 19305DP TCR. **a** IFN-γ production from 19305DP-TCR or CD8SP-TCR-transduced CD8^+^ T cells against wild-type or alanine-substituted NY-ESO-1_157-165_ peptides at different concentration was investigated by intracellular cytokine staining. **b** Percentage of relative IFN-γ production on TCR-transduced CD8^+^ T cells against alanine-substituted peptides at peptide concentration of 0.1 μM. Data are represented as % recognition as compared to % IFN-γ-producing CD8^+^ T cells against wild-type NY-ESO-1_157-165_ peptide. **c** 19305DP-TCR-transduced T cells were cocultured with the indicated panel of normal human tissue-derived primary cells or melanoma cell lines for 24 h. Some primary cell lines were pulsed with NY-ESO-1_157-165_ peptide (+pep) before coculture. IFN-γ level in the supernatant was measured by ELISA

IFN-γ production from TCR-transduced T cells that were stimulated by A*02^+^ EBV-B cells that were pulsed with alanine-substituted and wild-type peptides at concentrations ranged from 10 μM to 100 pM was determined by intracellular cytokine staining (Fig. 6a). Consistent with previous report [37], substitution of cysteine residue, which is located at the anchor position for HLA-A*02, with alanine significantly increased recognition by 19305DP-TCR and CD8SP-TCR. Key amino acid residues in NY-ESO-1_157-165_ epitope recognized by 19305DP-TCR were determined at suboptimal peptide concentration (0.1 μM) to recognize the wild-type epitope by 19305DP-TCR-transduced CD8^+^ T cells (Figs. 3b, d and 6a). Alanine substitution of most residues in the central region of the epitope LLMWIT completely inhibited recognition by 19305DP-TCR (Fig. 6b). In contrast, the recognition motif for CD8SP-TCR was determined to be LLMWxT, suggesting that 19305DP-TCR has more strict specificity on antigen recognition than CD8SP-TCR. There is no human protein containing LLMWIT based on in silico screening using the ScanProsite tool [38] except for NY-ESO-1 and its family member LAGE-1 which is also one of cancer-testis antigens. The second leucine residue in the NY-ESO-1_157-165_ epitope is an anchor residue to bind to A*02 and is not considered to be recognized by TCR. Removing this anchor residue and searching LMWIT returned only NY-ESO-1 and LAGE-1. Furthermore, 19305DP-TCR-transduced T cells did not show any reactivity against naturally A*02^+^ or A*02-transduced normal human tissue-derived primary cells from different tissue origins (Fig. 6c and Additional file 10). These results demonstrated the safety of 19305DP-TCR-transduced T cells, and thereby an excellent candidate for therapeutic translation for clinical use in patients due to its strict NY-ESO-1 specificity.

## Discussion

In this study, we have identified and characterized a naturally occurring CD4^+^CD8^+^ double-positive T-cell clone expressing CD4- and CD8-independent NY-ESO-1-specific TCR. 19305DP clone expressed a mixed gene profile for CD4^+^ and CD8^+^ T-cell clones, suggesting 19305DP is distinct from the single-positive CD4^+^ or CD8^+^ T-cell subset. We suggest that a comprehensive analysis of CD4^+^CD8^+^ double-positive T-cell clones in the periphery or tumor tissues would yield important insights into T-cell ontogeny and function. It is known that a small part of peripheral T cells can escape from thymic negative selection [15], although the mechanisms for such escape are largely unknown. The increase in the fraction of CD4^+^CD8^+^ double-positive T cells in the periphery has been discussed in terms of pathogenesis in autoimmune diseases or viral infection. In cancer, it has been reported that these double-positive T cells are enriched in the tumor microenvironment, and that tumor-infiltrating double-positive T cells recognize cancer cells although the specific antigen was not determined [39]. Therefore, our study is the first to define a bona fide tumor antigen recognized by CD4^+^CD8^+^ double-positive T cells in cancer patients.

In addition to αβ T cells that have escaped thymic deletion, co-expression of CD4 and CD8 molecules is found on a subset of unconventional T cells such as natural killer T (NKT) cells and intraepithelial T cells [32, 40]. In addition, it was reported that a subset of CD8^+^ T cells express CD4 molecules, or CD4^+^ T cells express CD8αα homodimers after activation or IL-4 signaling, respectively [41–43]. Although our characterization of 19305DP did not address ontogeny of this double-positive T cells, expression of lineage markers as well as extraordinarily high-affinity NY-ESO-1-specific TCR is in support of the origin of 19305DP as a rare population that escaped from thymic negative selection.

Importantly, recognition of cancer cells by 19305DP was not blocked by anti-CD8 mAb, indicating that this 19305DP-TCR was of high-affinity (Fig. 2d). In contrast, the same antibody completely blocked reactivity of conventional CD8^+^ T-cell clone recognizing the same epitope. Generally, TCR affinity can be increased by amino acid substitution in their antigen-binding domain [4, 44]. By comparing function of a series of mutant TCRs with different affinity, TCR affinity to provide optimal function was estimated to be below 4–5 μM. It will be interesting to measure affinity of naturally occurring 19305DP-TCR and other TCRs used in this study. Interestingly, avidity to recognize titrated peptides was comparable in CD8^+^ T cells when they were engineered to express TCRs from 19305DP and conventional CD8^+^ T cells (CD8SP), indicating that CD8-MHC interaction and/or CD8-signaling could sufficiently compensate lower affinity by tumor antigen-specific conventional CD8^+^ T cells (Fig. 3b).

Consistent with observations by other groups, our CD8-independent TCR from 19305DP provided strong tumor reactivity when it was expressed on CD4^+^ T cells in addition to CD8^+^ T cells. In anti-tumor immunity, CD8^+^ T cells have been considered as the main effector cells to destroy cancer targets, whereas the role of CD4^+^ T cells has been considered to be as helper of induction and maintenance of CD8^+^ T cells, as well as other immune cells such as antigen-presenting cells and B cells. As expected, only 19305DP-TCR-transduced CD8^+^ T cells showed strong cytotoxicity in vitro, although CD4^+^T cells significantly induced apoptosis of cancer cells after overnight coculture. In addition, 19305DP-TCR-transduced CD4^+^ T cells inhibited in vivo tumor growth at similar efficacy compared to CD8^+^ T cells engineered with the same TCR. Most current ACT clinical trials testing TCR gene-engineered T cells utilize polyclonally activated T cells to infuse products containing both CD4^+^ and CD8^+^ T cells. Therefore, utilizing a CD8-independent TCR gene rather than TCRs that are functional only in CD8^+^ T cells offers the potential for significantly higher clinical benefit by the direct anti-tumor effects of CD4^+^ T cells, and the collaborative anti-tumor effects by CD4^+^ and CD8^+^ T cells.

Although in vitro cytotoxic activity of CD4^+^ T cells was weaker than CD8^+^ T cells, in vivo anti-tumor activity of CD4^+^ T cells was similar to CD8^+^ T cells. Potent in vivo anti-tumor efficacy of TCR gene-engineered CD4^+^ T cells was demonstrated using MHC class I-restricted tyrosinase-specific T cells in immunocompetent mice by Frankel et al. [7] In our human tumor xenograft models in immunodeficient NSG mice, there is no endogenous tumor-reactive CD8^+^ T cells that could be activated by CD4^+^ T cells and collaboratively suppress tumor growth, suggesting that 19305DP-TCR-transduced CD4^+^ T cells directly inhibited tumor growth. Because the parental 19305DP clone expressed lower CD8 molecules compared with CD8^+^ single-positive T-cell clones, 19305DP-TCR expressed on fully functional CD8^+^ T cells may cross-react to antigens expressed in normal tissues. In this case, 19305DP-TCR-transduced CD4^+^ T cells may be a safer approach for assessing function and potential side effects in clinical trials, although the process of isolating engineered CD4^+^ T cells for transduction could add additional complexity to cell manufacturing. As we previously demonstrated, in vitro and in vivo tumor growth was significantly inhibited by MHC class II-restricted TR-CD4 cells, at least partially through IFN-γ production [26]. The in vivo mechanism(s) by which these MHC class I-restricted CD4^+^ T cells mediate anti-tumor effects could be further investigated by in vivo blockade of IFN-γ, TNF-α or IL-2, or by using IFN-γR or TNF-αR knockdown cancer cells.

It is noteworthy that 19305DP-TCR-transduced CD4^+^ T cells recognize cancer cells in an MHC class I-restricted manner, in contrast to MHC class II-restriction of conventional CD4^+^ T cells. Therefore, it would be important to investigate whether MHC class I-restricted CD4^+^ T cells are sufficient to support anti-tumor immunity, or whether physiological MHC class II-restricted CD4^+^ T cells would play distinct roles in the presence or absence of MHC class II^+^ antigen-presenting cells. In this regard, we have characterized MHC class II-restricted CD4^+^ T cells that have potent anti-tumor activity by direct recognition of human cancer cells in a MHC class II-restricted manner [26]. Using TCR gene-transduced CD4^+^ T cells, we are currently preparing experiments comparing MHC class I- and MHC class II-restricted CD4^+^ T cells using human HLA-transgenic NSG mice.

The possibility that 19305DP was not negatively selected in thymic selection may raise the likelihood that 19305DP-TCR could cross-react with other antigens expressed in normal tissues. The patient from whom 19305DP was established was HLA-B*27^+^ and had a history of ankylosing spondylitis, an autoimmune disease strongly associated with HLA-B*27 antigen. Interestingly, this patient presented with advanced stage IIIc ovarian cancer, and exhibited remarkable long-term survival, with no evidence of disease recurrence for more than 5 years following completion of standard surgery and chemotherapy for her disease. Because involvement of 19305DP clone in the pathogenesis of her autoimmune disease was not completely excluded, we carefully investigated the specificity of 19305DP-TCR by determining TCR-recognizing motif and screening human proteins that share the motif. Several strategies to test cross-reactivity of TCR genes have been developed such as testing reactivity against a panel of various normal human tissue-derived cells and/or against homologous proteins that share TCR recognition motifs that could be identified by alanine substitution of the synthetic epitope peptides [45]. Alanine substitution experiments demonstrated that most (5/9) residues (LMWIT) in the epitope are required in the interaction with TCR and in silico screening showed that only NY-ESO-1 and LAGE-1 have this motif. Finally, we demonstrated that 19305DP-TCR-transduced T cells show no reactivity against a panel of normal human tissue-derived primary cells from different tissue origins. Collectively, our data support the conclusion that 19305DP-TCR is only reactive against A*02^+^NY-ESO-1^+^/LAGE-1^+^ cancer cells with no cross-reactivity against any proteins expressed in humans. Nevertheless, because no in vitro or in silico testing can reliably determine reactivity against all proteins expressed in humans, safety of 19305DP-TCR-engineered T cells should be determined by careful dose-escalation study in clinical trials

## Conclusions

In summary, we have identified a unique tumor antigen-specific CD4^+^CD8^+^ T-cell subset which expresses a CD8-independent tumor antigen-specific TCR. Our observations indicate that selecting tumor antigen-specific TCR genes from CD4^+^CD8^+^ double-positive T cells could be an alternative strategy for discovering high-affinity tumor antigen-specific TCR genes. This unique approach obviates the need for affinity enhancement, which minimizes the risk of lethal off-target toxicities that have occurred in some clinical trials [12, 13]. Given that these CD4^+^CD8^+^ double positive αβ T cells are rare because of thymic negative selection, our study is based on a single such clone, but the principles could be extended to other such clones and antigen targets. Due to its comparable tumor reactivity with affinity-matured or murine TCRs and potent anti-tumor effects, our 19305DP-TCR can be considered an ideal therapeutic TCR gene product for manufacturing engineered T cells for ACT in A*02^+^ patients with NY-ESO-1-expressing tumors.

## Additional files

Additional file 1: Nanostring analyses of mRNA expression of T-cell clones with or without TCR stimulation. Indicated T-cell clones were stimulated with immorbilized anti-CD3 antibody for 2 h (αCD3) or unstimulated. Expression level of mRNA was determined using Nanostring system. Clustering analyses of normalized expression data were performed by nSolver Analysis Software. (PDF 148 kb)

Additional file 2: Intensity of HLA-A2 and NY-ESO-1 expression on A*02^+^NY-ESO-1^+^ cancer cell lines. Surface HLA-A2 (clone: BB7.2) and cytoplasmic NY-ESO-1 (clone: 219–510) expression was analyzed by flow cytometry. Shaded histogram is unstained control. (PDF 144 kb)

Additional file 3: TCR α and β chain nucleotide sequences of 19305DP. (PDF 24 kb)

Additional file 4: Generation of TCR gene-transduced T cells. (A) Schematic representation of retroviral TCR expression vector for 19305DP- and CD8SP-TCR. LTR: long terminal repeats; *ѱ+*: extended packaging signal; *SA*: Splice acceptor site from the first intron-exon junction of human elongation factor-1α; VDJβ: TCR β chain variable-diverse-joining regions; Cβ: TCR β chain constant region; T2A: SGSG-linker connected to the T2A translational skipping sequence; VJα: TCR α chain variable-joining regions; Cα: TCR α chain constant region. (B) Transduction efficiency of 19305DP-TCR (Vβ8) and CD8SP-TCR (Vβ3) gene-engineered T cells was determined by flow cytometry using corresponding anti-Vβ subtype-specific antibodies and A*02/NY-ESO-1_157-165_ tetramer. (PDF 223 kb)

Additional file 5: Tumor recognition of TCR gene-transduced T cells against cancer cell lines. Percentages of IFN-γ producing cells in CD8^+^ and CD4^+^ T cells against a panel of melanoma and ovarian cancer cell lines were determined by intracellular cytokine staining. Pooled data from two independent experiments were shown. (PDF 94 kb)

Additional file 6: Effect of co-ligation signals on recognition of cancer cells by TCR-transduced T cells. Reactivity of 19305DP-TCR or CD8SP-TCR-transduced T cells against A375 or Mel624.38 was tested by intracellular cytokine staining. Before coculture, cancer cells or T cells were incubated with or without (−) anti-MHC class I (αHLA-A,B,C), anti-CD4 (αCD4) or anti-CD8 (αCD8) antibody for 30 min and then T cells or cancer cells were added without washing out the antibodies. Percentages of IFN-γ producing CD4^+^ or CD8^+^ T cells were plotted from two independent experiments. (PDF 92 kb)

Additional file 7: Comparison of off-target reactivity of 19305DP-TCR and other high-affinity TCRs. Recognition of NY-ESO-1^-^ or HLA-A2^-^ melanoma cell lines (SK-MEL-29: A*02^+^NY-ESO-1^-^; Mel888: A*02^-^NY-ESO-1^-^; Mel938: A*02^-^NY-ESO-1^+^) by 19305DP-TCR-transduced T cells was compared to T cells transduced with affinity-enhanced TCR (LY) or murine TCR (mTCR) by intracellular cytokine staining. (PDF 287 kb)

Additional file 8: Transduction efficiency of TCR in isolated CD4^+^ and CD8^+^ T cells. CD8^+^ T cells or CD4^+^ T cells were depleted from normal donor PBMC and infected with retroviral vector for 19305DP-TCR (Vβ8) or CD8SP-TCR (Vβ3). Transduction efficiency and CD4/CD8 purity was investigated by flow cytometry using corresponding Vβ-subtype-specific antibodies and anti-CD8 antibody. (PDF 199 kb)

Additional file 9: Cytokine and cytotoxic molecule production from TCR-transduced T cells. Whole PBMC, CD4^+^ or CD8^+^ T cells that were transduced with 19305DP-TCR or CD8SP-TCR gene were cocultured with A375 and the culture supernatant was harvested at day 1 - day 4. TNF-α, IL-2, granzyme B (Gzm B) and perforin levels in the culture supernatant were measured by ELISA. (PDF 100 kb)

Additional file 10: Expression of HLA-A2 and HLA class I on A*02^+^ or A*02-transduced normal cell lines. Surface HLA-A2 and HLA class I (HLA-A,B,C, clone: W6/32) expression was analyzed by flow cytometry. (PDF 169 kb)

## Abbreviations

A*02: HLA-A*02:01
ACT: Adoptive cell therapy
CD4SP: CD4^+^ single-positive T-cell clone
CD8SP: CD8^+^ single-positive T-cell clone
Cw*03: HLA-Cw*03:04
DP*04: HLA-DP*04:01
DR*01: HLA-DR*01:01
EBV: Epstein-Barr virus
LY: a95:LY/c259 TCR
mTCR: murine TCR
NCI: National Cancer Institute
NSG: NOD/SCID/IL-2Rγ-deficient
PBMC: Peripheral blood mononuclear cells
PHA: Phytohemagglutinin
PI: Propidium iodide
TCR: T-cell receptor
TR-CD4: Tumor-recognizing CD4^+^ T cells
