# Supplementary material for: A rare population of tumor antigen-specific CD4+CD8+ double-positive αβ T lymphocytes uniquely provide CD8-independent TCR genes for engineering therapeutic T cells
Source: J Immunother Cancer. 2019 Jan 9;7:7. doi: 10.1186/s40425-018-0467-y (PMC6325755; doi:10.1186/s40425-018-0467-y)
Supplement: Supplementary file 1 — Nanostring analyses of mRNA expression of T-cell clones with or without TCR stimulation. Indicated T-cell clones were stimulated with immorbilized anti-CD3 antibody for 2 h (αCD3) or unstimulated. Expression level of mRNA was determined using Nanostring system. Clustering analyses of normalized expression data were performed by nSolver Analysis Software. (PDF 148 kb) [file 40425_2018_467_MOESM1_ESM.pdf]

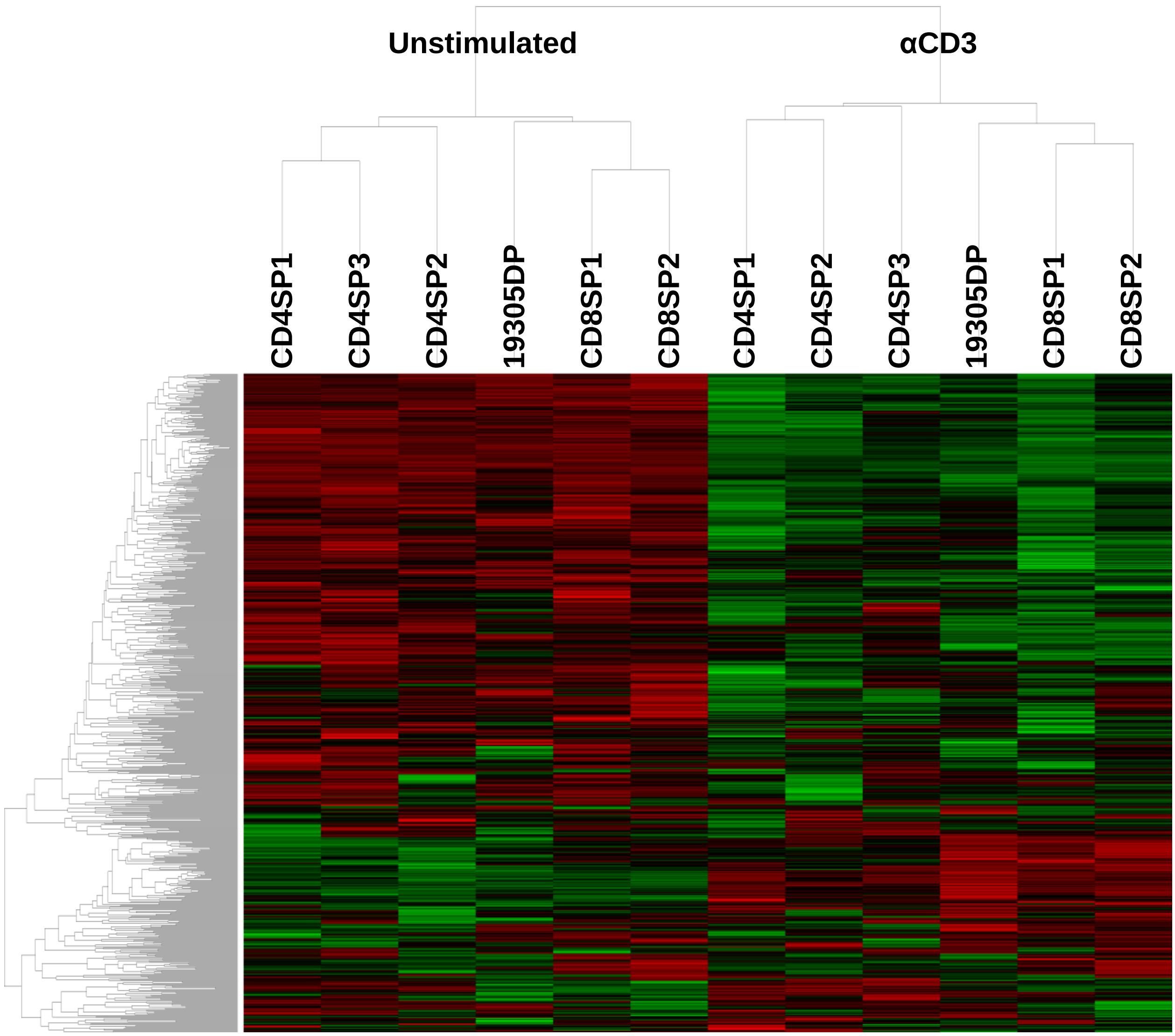

Additional file 1: Nanostring analyses of mRNA expression of T-cell clones with or without TCR stimulation. Indicated T-cell clones were stimulated with immorbilized anti-CD3 antibody for 2 hours (αCD3) or unstimulated. Expression level of mRNA was determined using Nanostring system. Clustering analyses of normalized expression data were performed by nSolver Analysis Software.
