# Supplementary material for: A rare population of tumor antigen-specific CD4+CD8+ double-positive αβ T lymphocytes uniquely provide CD8-independent TCR genes for engineering therapeutic T cells
Source: J Immunother Cancer. 2019 Jan 9;7:7. doi: 10.1186/s40425-018-0467-y (PMC6325755; doi:10.1186/s40425-018-0467-y)
Supplement: Supplementary file 2 — Intensity of HLA-A2 and NY-ESO-1 expression on A*02+NY-ESO-1+ cancer cell lines. Surface HLA-A2 (clone: BB7.2) and cytoplasmic NY-ESO-1 (clone: 219–510) expression was analyzed by flow cytometry. Shaded histogram is unstained control. (PDF 144 kb) [file 40425_2018_467_MOESM2_ESM.pdf]

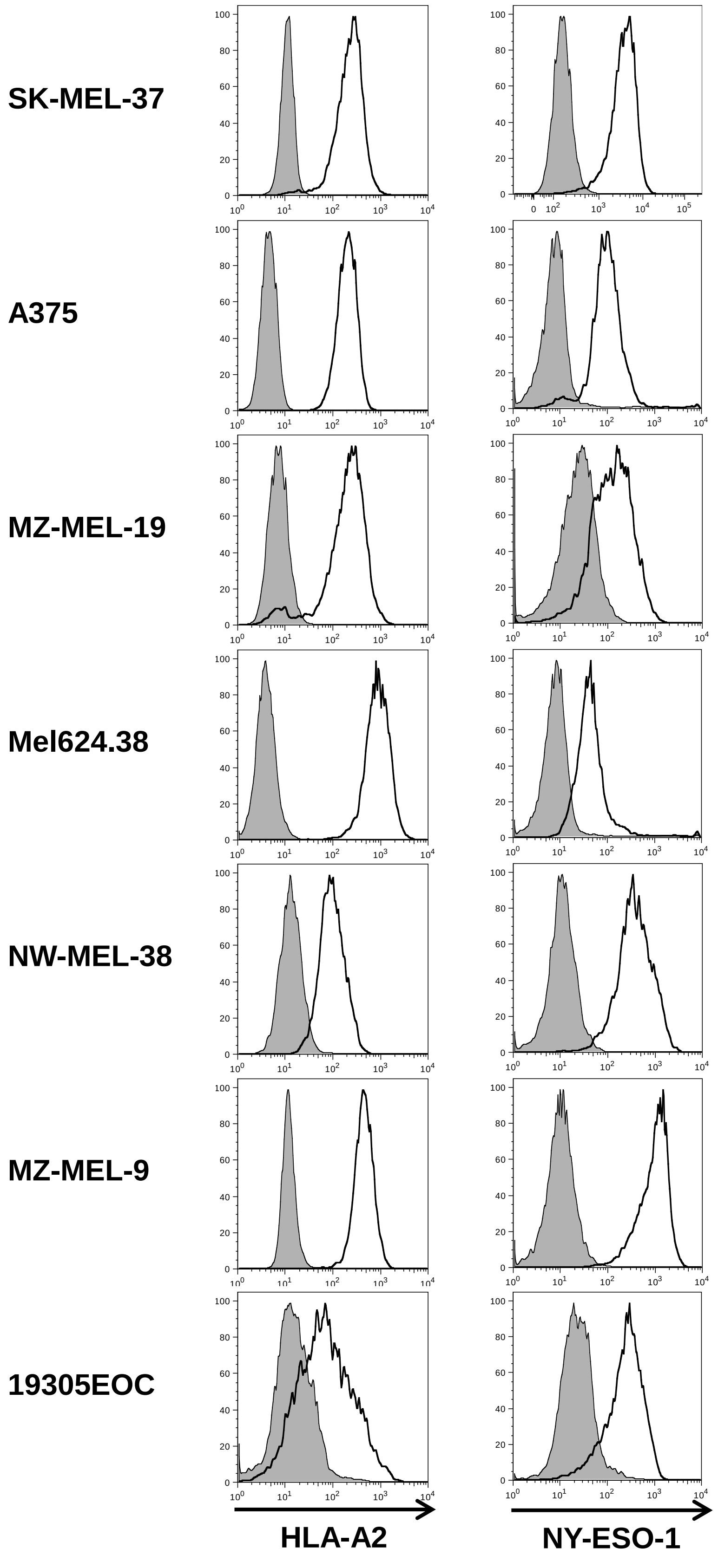

Additional file 2. Intensity of HLA-A2 and NY-ESO-1 expression on A*02⁺NY-ESO-1⁺ cancer cell lines. Surface HLA-A2 (clone: BB7.2) and cytoplasmic NY-ESO-1 (clone: 219-510) expression was analyzed by flow cytometry. Shaded histogram is unstained control.
