# Supplementary material for: A rare population of tumor antigen-specific CD4+CD8+ double-positive αβ T lymphocytes uniquely provide CD8-independent TCR genes for engineering therapeutic T cells
Source: J Immunother Cancer. 2019 Jan 9;7:7. doi: 10.1186/s40425-018-0467-y (PMC6325755; doi:10.1186/s40425-018-0467-y)
Supplement: Supplementary file 3 — TCR α and β chain nucleotide sequences of 19305DP. (PDF 24 kb) [file 40425_2018_467_MOESM3_ESM.pdf]

[TCR α chain]
ATGGAAACTCTCCTGGGAGTGTCTTTGGTGATTCTATGGCTTCAACTGGCTAGGGTGAA
CAGTCAACAGGGAGAAGAGGATCCTCAGGCCTTGAGCATCCAGGAGGGTGAAAATGCC
ACCATGAACTGCAGTTACAAAACTAGTATAAACAATTTACAGTGGTATAGACAAAATTCA
GGTAGAGGCCTTGTCCACCTAATTTTAATACGTTCAAATGAAAGAGAGAAACACAGTGG
AAGATTAAGAGTCACGCTTGACACTTCCAAGAAAAGCAGTTCCTTGTTGATCACGGCTTC
CCGGGCAGCAGACACTGCTTCTTACTTCTGTGCTACGGACGGGGGGGGCACCCTCACC
TTTGGGAAGGGGACTATGCTTCTAGTCTCTCCAGATATCCAGAACCCTGACCCTGCCGT
GTACCAGCTGAGAGACTCTAAATCCAGTGACAAGTCTGTCTGCCTATTCACCGATTTTGA
TTCTCAAACAAATGTGTCACAAAGTAAGGATTCTGATGTGTATATCACAGACAAAACTGT
GCTAGACATGAGGTCTATGGACTTCAAGAGCAACAGTGCTGTGGCCTGGAGCAACAAAT
CTGACTTTGCATGTGCAAACGCCTTCAACAACAGCATTATTCCAGAAGACACCTTCTTCC
CCAGCCCAGAAAGTTCCTGTGATGTCAAGCTGGTCGAGAAAAGCTTTGAAACAGATACG
AACCTAAACTTTCAAAACCTGTCAGTGATTGGGTTCCGAATCCTCCTCCTGAAAGTGGC
CGGGTTTAATCTGCTCATGACGCTGCGGCTGTGGTCCAGCTGA

[TCR β chain]
ATGGACTCCTGGACCCTCTGCTGTGTGTCCCTTTGCATCCTGGTAGCAAAGCACACAGA
TGCTGGAGTTATCCAGTCACCCCGGCACGAGGTGACAGAGATGGGACAAGAAGTGACT
CTGAGATGTAAACCAATTTCAGGACACGACTACCTTTTCTGGTACAGACAGACCATGAT
GCGGGGACTGGAGTTGCTCATTTACTTTAACAACAACGTTCCGATAGATGATTCAGGGA
TGCCCGAGGATCGATTCTCAGCTAAGATGCCTAATGCATCATTCTCCACTCTGAAGATC
CAGCCCTCAGAACCCAGGGACTCAGCTGTGTACTTCTGTGCCAGCAAGTGGGGCGGCA
CTGAAGCTTTCTTTGGACAAGGCACCAGACTCACAGTTGTAGAGGACCTGAACAAGGTG
TTCCCACCCGAGGTCGCTGTGTTTGAGCCATCAGAAGCAGAGATCTCCCACACCCAAAA
GGCCACACTGGTGTGCCTGGCCACAGGCTTCTTCCCTGACCACGTGGAGCTGAGCTGG
TGGGTGAATGGGAAGGAGGTGCACAGTGGGGTCAGCACGGACCCGCAGCCCCTCAAG
GAGCAGCCCGCCCTCAATGACTCCAGATACTGCCTGAGCAGCCGCCTGAGGGTCTCGG
CCACCTTCTGGCAGAACCCCCGCAACCACTTCCGCTGTCAAGTCCAGTTCTACGGGCT
CTCGGAGAATGACGAGTGGACCCAGGATAGGGCCAAACCCGTCACCCAGATCGTCAGC
GCCGAGGCCTGGGGTAGAGCAGACTGTGGCTTTACCTCGGTGTCCTACCAGCAAGGG
GTCCTGTCTGCCACCATCCTCTATGAGATCCTGCTAGGGAAGGCCACCCTGTATGCTGT
GCTGGTCAGCGCCCTTGTGTTGATGGCCATGGTCAAGAGAAAGGATTTCTGA

Additional file 3. TCR α and β chain nucleotide sequences of 19305DP.
