# Supplementary material for: A rare population of tumor antigen-specific CD4+CD8+ double-positive αβ T lymphocytes uniquely provide CD8-independent TCR genes for engineering therapeutic T cells
Source: J Immunother Cancer. 2019 Jan 9;7:7. doi: 10.1186/s40425-018-0467-y (PMC6325755; doi:10.1186/s40425-018-0467-y)
Supplement: Supplementary file 4 — Generation of TCR gene-transduced T cells. (A) Schematic representation of retroviral TCR expression vector for 19305DP- and CD8SP-TCR. LTR: long terminal repeats; ѱ+: extended packaging signal; SA: Splice acceptor site from the first intron-exon junction of human elongation factor-1α; VDJβ: TCR β chain variable-diverse-joining regions; Cβ: TCR β chain constant region; T2A: SGSG-linker connected to the T2A translational skipping sequence; VJα: TCR α chain variable-joining regions; Cα: TCR α chain constant region. (B) Transduction efficiency of 19305DP-TCR (Vβ8) and CD8SP-TCR (Vβ3) gene-engineered T cells was determined by flow cytometry using corresponding anti-Vβ subtype-specific antibodies and A*02/NY-ESO-1157-165 tetramer. (PDF 223 kb) [file 40425_2018_467_MOESM4_ESM.pdf]

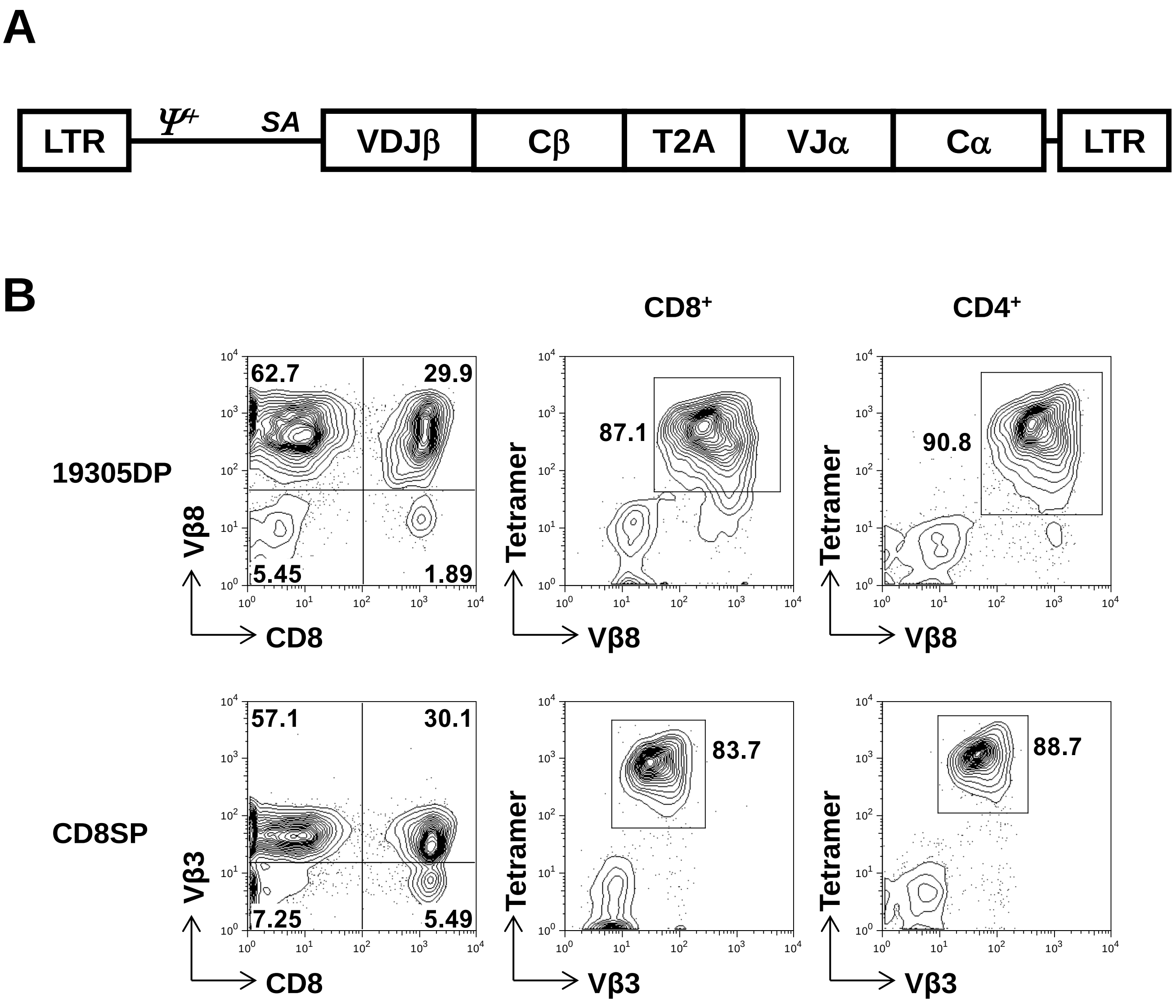

Additional file 4: Generation of TCR gene-transduced T cells. (A) Schematic representation of retroviral TCR expression vector for 19305DP- and CD8SP-TCR. LTR: long terminal repeats; ψ^{+}: extended packaging signal; *SA*: Splice acceptor site from the first intron-exon junction of human elongation factor-1α; VDJβ: TCR β chain variable-diverse-joining regions; Cβ: TCR β chain constant region; T2A: SGSG-linker connected to the T2A translational skipping sequence; VJα: TCR α chain variable-joining regions; Cα: TCR α chain constant region. (B) Transduction efficiency of 19305DP-TCR (Vβ8) and CD8SP-TCR (Vβ3) gene-engineered T cells was determined by flow cytometry using corresponding anti-Vβ subtype-specific antibodies and A*02/NY-ESO-$1_{157\text{-}165}$ tetramer.
