# Supplementary material for: A rare population of tumor antigen-specific CD4+CD8+ double-positive αβ T lymphocytes uniquely provide CD8-independent TCR genes for engineering therapeutic T cells
Source: J Immunother Cancer. 2019 Jan 9;7:7. doi: 10.1186/s40425-018-0467-y (PMC6325755; doi:10.1186/s40425-018-0467-y)
Supplement: Supplementary file 5 — Tumor recognition of TCR gene-transduced T cells against cancer cell lines. Percentages of IFN-γ producing cells in CD8+ and CD4+ T cells against a panel of melanoma and ovarian cancer cell lines were determined by intracellular cytokine staining. Pooled data from two independent experiments were shown. (PDF 94 kb) [file 40425_2018_467_MOESM5_ESM.pdf]

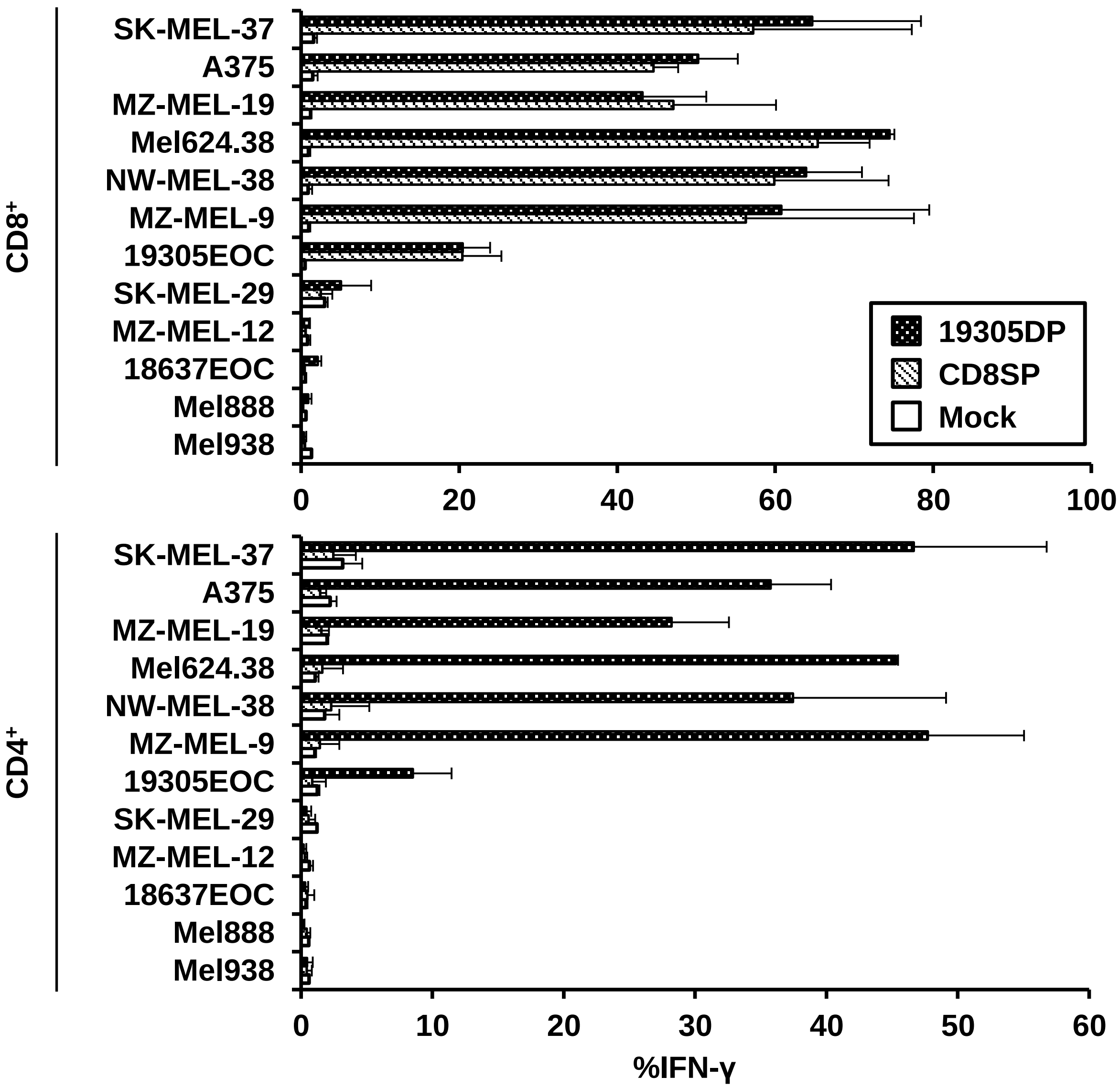

Additional file 5: Tumor recognition of TCR gene-transduced T cells against cancer cell lines. Percentages of IFN-γ producing cells in $CD8^+$ and $CD4^+$ T cells against a panel of melanoma and ovarian cancer cell lines were determined by intracellular cytokine staining. Pooled data from two independent experiments were shown.
