# Supplementary material for: A rare population of tumor antigen-specific CD4+CD8+ double-positive αβ T lymphocytes uniquely provide CD8-independent TCR genes for engineering therapeutic T cells
Source: J Immunother Cancer. 2019 Jan 9;7:7. doi: 10.1186/s40425-018-0467-y (PMC6325755; doi:10.1186/s40425-018-0467-y)
Supplement: Supplementary file 6 — Effect of co-ligation signals on recognition of cancer cells by TCR-transduced T cells. Reactivity of 19305DP-TCR or CD8SP-TCR-transduced T cells against A375 or Mel624.38 was tested by intracellular cytokine staining. Before coculture, cancer cells or T cells were incubated with or without (−) anti-MHC class I (αHLA-A,B,C), anti-CD4 (αCD4) or anti-CD8 (αCD8) antibody for 30 min and then T cells or cancer cells were added without washing out the antibodies. Percentages of IFN-γ producing CD4+ or CD8+ T cells were plotted from two independent experiments. (PDF 92 kb) [file 40425_2018_467_MOESM6_ESM.pdf]

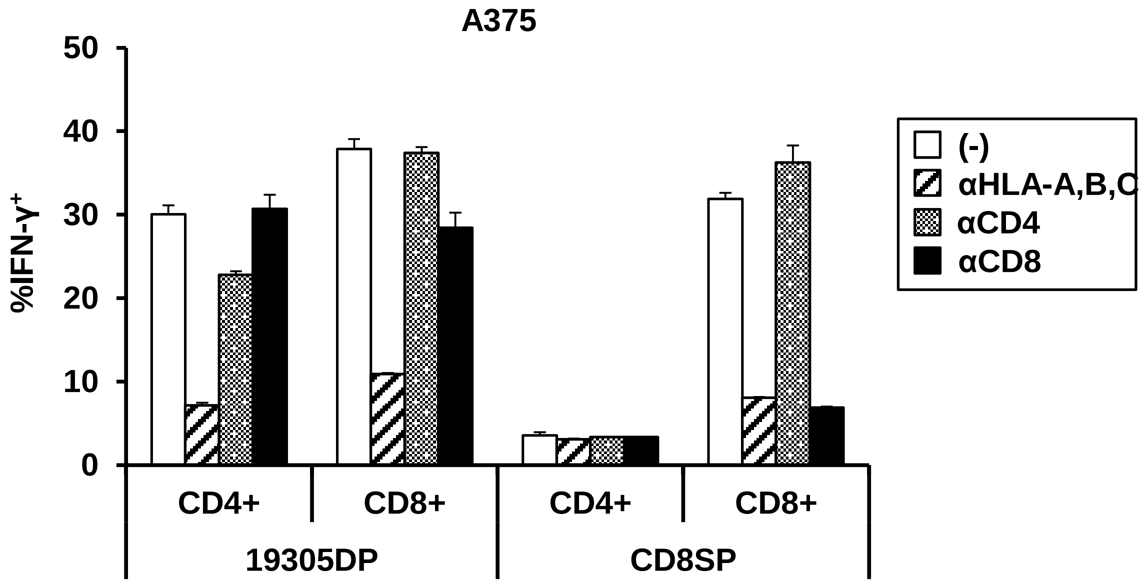

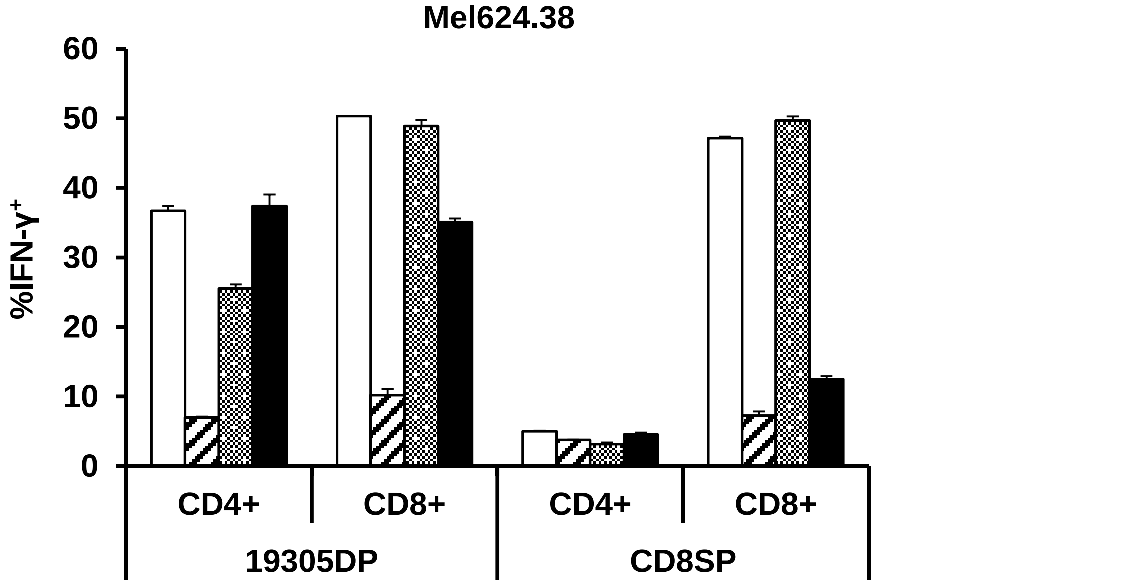

Additional file 6: Effect of co-ligation signals on recognition of cancer cells by TCR-transduced T cells. Reactivity of 19305DP-TCR or CD8SP-TCR-transduced T cells against A375 or Mel624.38 was tested by intracellular cytokine staining. Before coculture, cancer cells or T cells were incubated with or without (-) anti-MHC class I (αHLA-A,B,C), anti-CD4 (αCD4) or anti-CD8 (αCD8) antibody for 30 minutes and then T cells or cancer cells were added without washing out the antibodies. Percentages of IFN-γ producing $CD4^+$ or $CD8^+$ T cells were plotted from two independent experiments.
