# Supplementary material for: A rare population of tumor antigen-specific CD4+CD8+ double-positive αβ T lymphocytes uniquely provide CD8-independent TCR genes for engineering therapeutic T cells
Source: J Immunother Cancer. 2019 Jan 9;7:7. doi: 10.1186/s40425-018-0467-y (PMC6325755; doi:10.1186/s40425-018-0467-y)
Supplement: Supplementary file 7 — Comparison of off-target reactivity of 19305DP-TCR and other high-affinity TCRs. Recognition of NY-ESO-1- or HLA-A2- melanoma cell lines (SK-MEL-29: A*02+NY-ESO-1-; Mel888: A*02-NY-ESO-1-; Mel938: A*02-NY-ESO-1+) by 19305DP-TCR-transduced T cells was compared to T cells transduced with affinity-enhanced TCR (LY) or murine TCR (mTCR) by intracellular cytokine staining. (PDF 287 kb) [file 40425_2018_467_MOESM7_ESM.pdf]

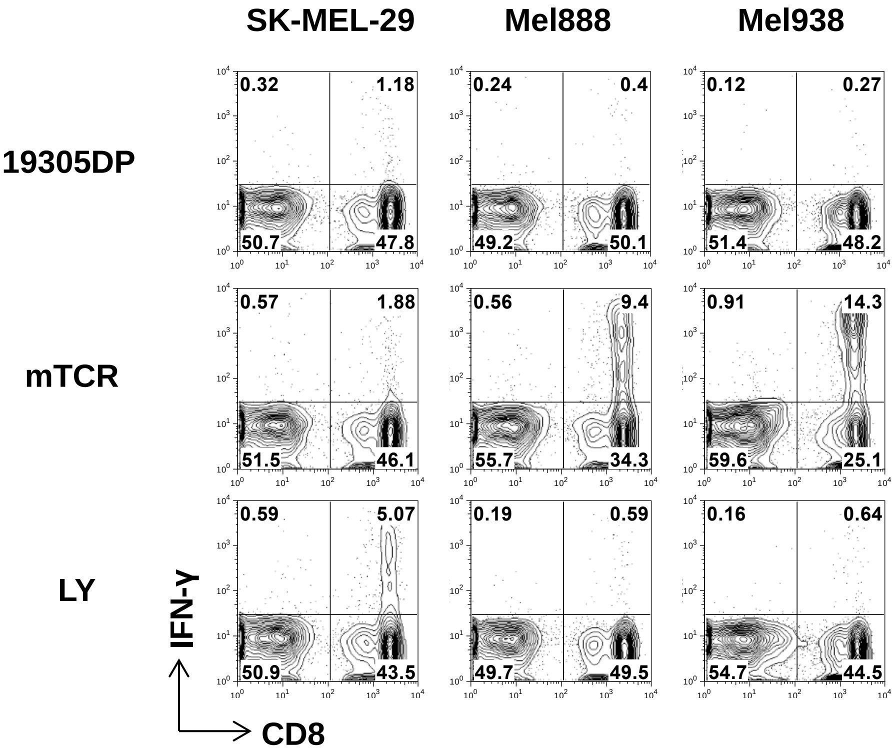

Additional file 7: Comparison of off-target reactivity of 19305DP-TCR and other high-affinity TCRs. Recognition of NY-ESO-1$^-$ or HLA-A2$^-$ melanoma cell lines (SK-MEL-29: A*02$^+$NY-ESO-1$^-$; Mel888: A*02$^-$NY-ESO-1$^-$; Mel938: A*02$^-$NY-ESO-1$^+$) by 19305DP-TCR-transduced T cells was compared to T cells transduced with affinity-enhanced TCR (LY) or murine TCR (mTCR) by intracellular cytokine staining.
