# Supplementary material for: A rare population of tumor antigen-specific CD4+CD8+ double-positive αβ T lymphocytes uniquely provide CD8-independent TCR genes for engineering therapeutic T cells
Source: J Immunother Cancer. 2019 Jan 9;7:7. doi: 10.1186/s40425-018-0467-y (PMC6325755; doi:10.1186/s40425-018-0467-y)
Supplement: Supplementary file 8 — Transduction efficiency of TCR in isolated CD4+ and CD8+ T cells. CD8+ T cells or CD4+ T cells were depleted from normal donor PBMC and infected with retroviral vector for 19305DP-TCR (Vβ8) or CD8SP-TCR (Vβ3). Transduction efficiency and CD4/CD8 purity was investigated by flow cytometry using corresponding Vβ-subtype-specific antibodies and anti-CD8 antibody. (PDF 199 kb) [file 40425_2018_467_MOESM8_ESM.pdf]

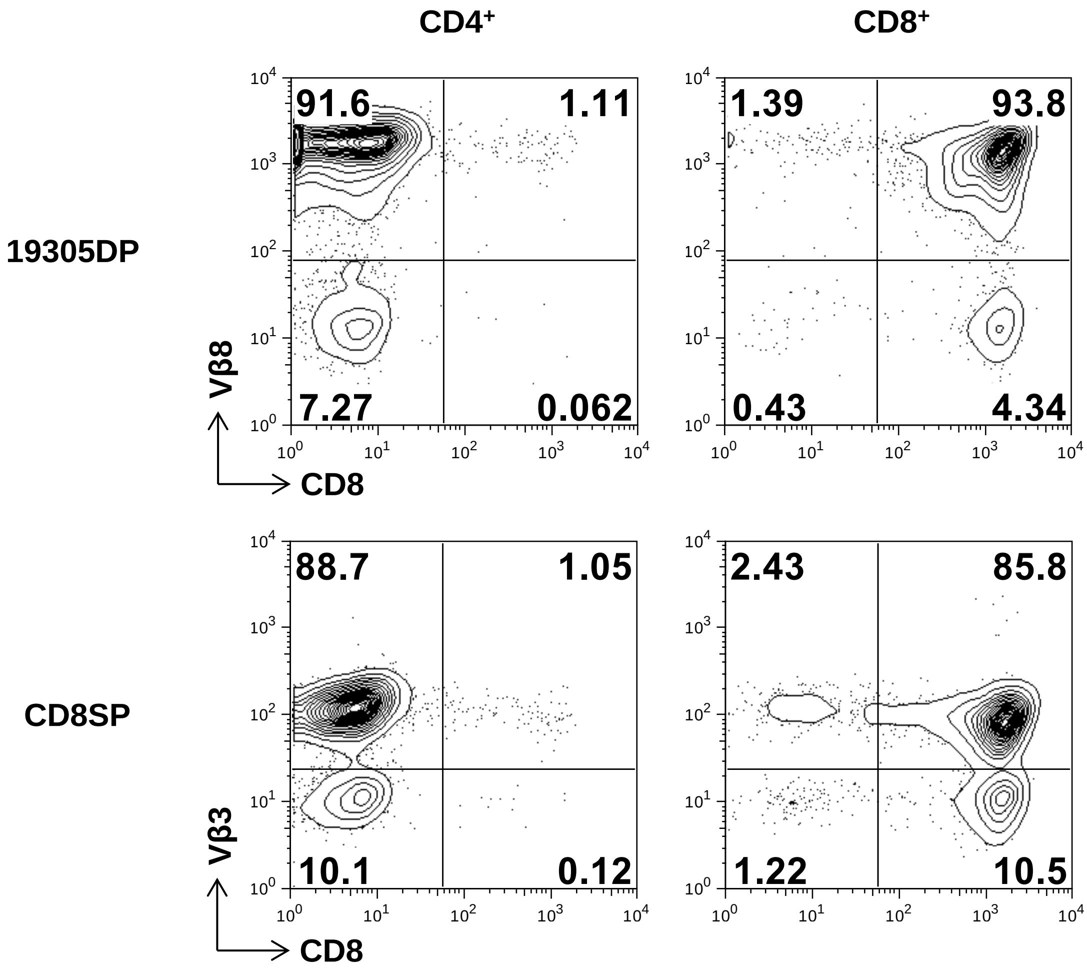

Additional file 8: Transduction efficiency of TCR in isolated CD4+ and CD8+ T cells. CD8+ T cells or CD4+ T cells were depleted from normal donor PBMC and infected with retroviral vector for 19305DP-TCR (Vβ8) or CD8SP-TCR (Vβ3). Transduction efficiency and CD4/CD8 purity was investigated by flow cytometry using corresponding Vβ-subtype-specific antibodies and anti-CD8 antibody.
