# Supplementary material for: A rare population of tumor antigen-specific CD4+CD8+ double-positive αβ T lymphocytes uniquely provide CD8-independent TCR genes for engineering therapeutic T cells
Source: J Immunother Cancer. 2019 Jan 9;7:7. doi: 10.1186/s40425-018-0467-y (PMC6325755; doi:10.1186/s40425-018-0467-y)
Supplement: Supplementary file 9 — Cytokine and cytotoxic molecule production from TCR-transduced T cells. Whole PBMC, CD4+ or CD8+ T cells that were transduced with 19305DP-TCR or CD8SP-TCR gene were cocultured with A375 and the culture supernatant was harvested at day 1 - day 4. TNF-α, IL-2, granzyme B (Gzm B) and perforin levels in the culture supernatant were measured by ELISA. (PDF 100 kb) [file 40425_2018_467_MOESM9_ESM.pdf]

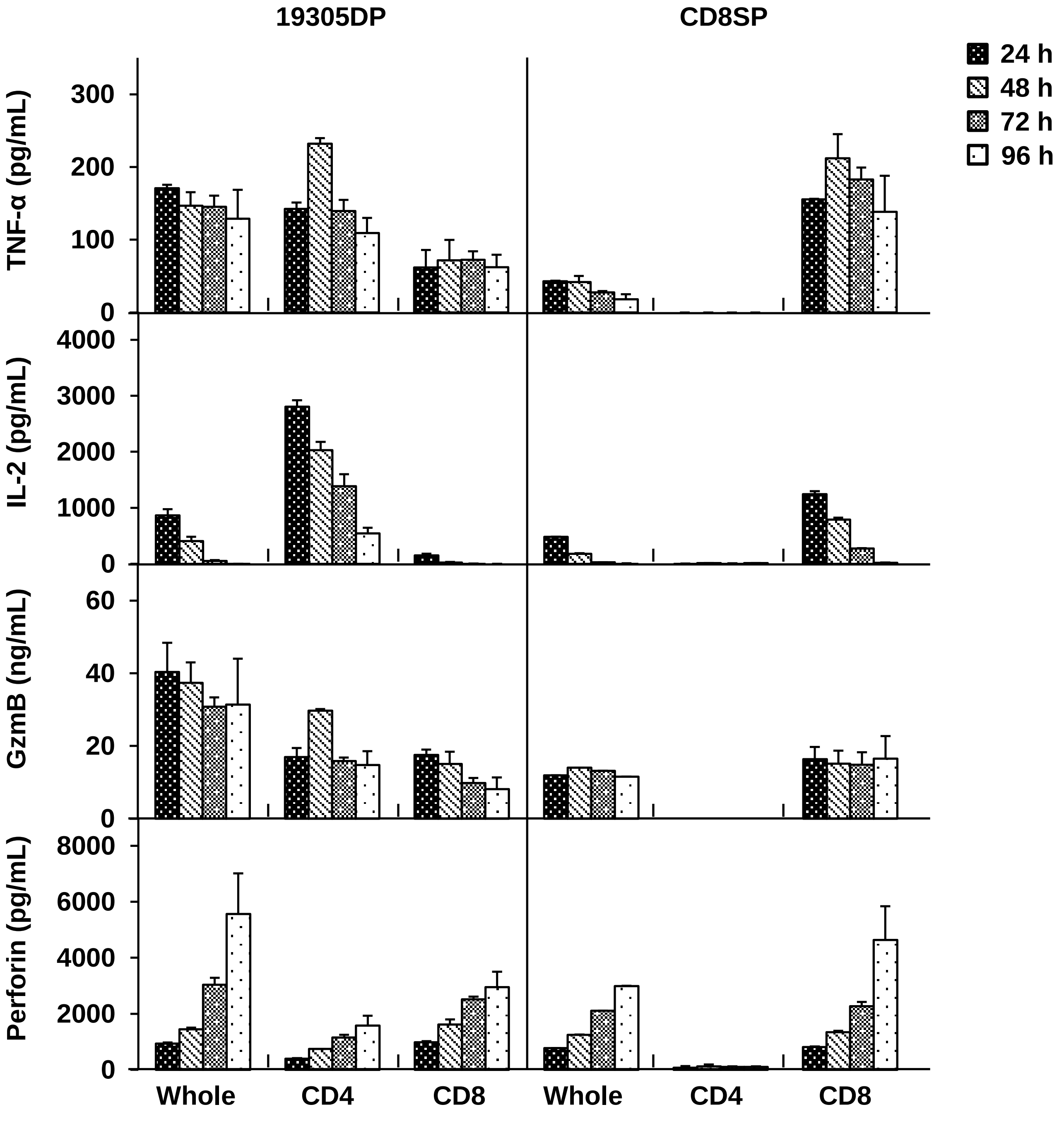

Additional file 9: Cytokine and cytotoxic molecule production from TCR-transduced T cells. Whole PBMC, $CD4^+$ or $CD8^+$ T cells that were transduced with 19305DP-TCR or CD8SP-TCR gene were cocultured with A375 and the culture supernatant was harvested at day 1 - day 4. TNF-α, IL-2, granzyme B (Gzm B) and perforin levels in the culture supernatant were measured by ELISA.
