# Supplementary material for: A rare population of tumor antigen-specific CD4+CD8+ double-positive αβ T lymphocytes uniquely provide CD8-independent TCR genes for engineering therapeutic T cells
Source: J Immunother Cancer. 2019 Jan 9;7:7. doi: 10.1186/s40425-018-0467-y (PMC6325755; doi:10.1186/s40425-018-0467-y)
Supplement: Supplementary file 10 — Expression of HLA-A2 and HLA class I on A*02+ or A*02-transduced normal cell lines. Surface HLA-A2 and HLA class I (HLA-A,B,C, clone: W6/32) expression was analyzed by flow cytometry. (PDF 169 kb) [file 40425_2018_467_MOESM10_ESM.pdf]

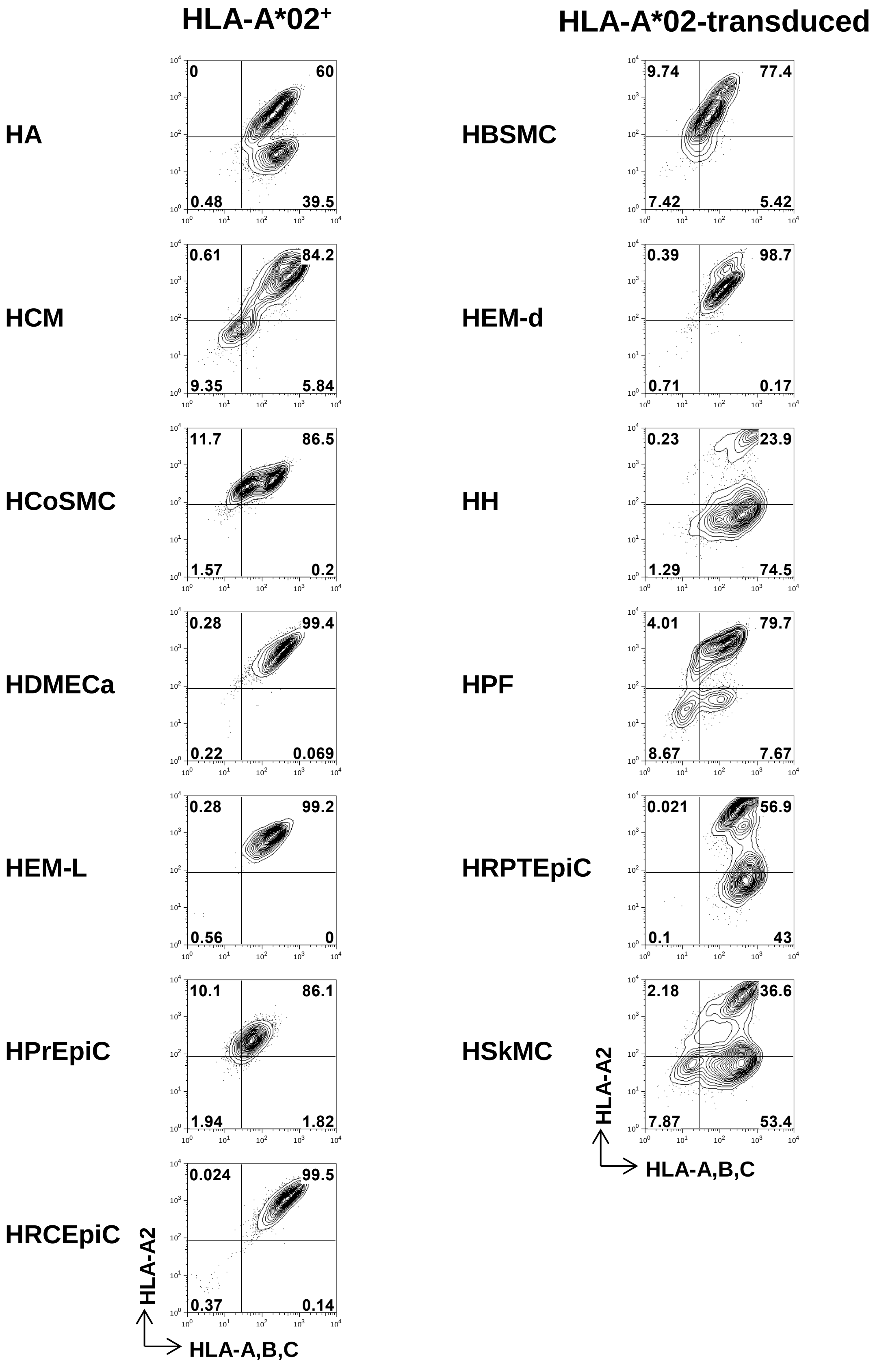

Additional file 10: Expression of HLA-A2 and HLA class I on A*02+ or A*02-transduced normal cell lines. Surface HLA-A2 and HLA class I (HLA-A,B,C, clone: W6/32) expression was analyzed by flow cytometry.
